# Supplementary material for: From symptom onset to treatment initiation: protocol for a narrative study exploring the journey of older adults with tuberculosis in the English Midlands, UK
Source: BMJ Open. 2023 Nov 17;13(11):e070933. doi: 10.1136/bmjopen-2022-070933 (PMC10660672; doi:10.1136/bmjopen-2022-070933)
Supplement: Supplementary data [file bmjopen-2022-070933supp001.pdf]

Supplementary Material

From symptom onset to treatment initiation: A narrative study of Older Adults with Tuberculosis (OAT)

The OAT Study

Topic Guide

(Date 20/09/2020 Version 1.0)

The OAT Study will take a narrative approach to data gathering. The guide will the trialled on English and non-English speaking participants and adapted if needed.

Step One: Introductions, consent and identity confirmation

- Introduce self and confirm that the call is to take consent and carry out the interview.
- Ask participant to confirm their name and date of birth.
- Confirm that this is a suitable time.
- Ask participant if they have any questions about the information sheets, consent form or study in general.
- Thanks for agreeing to participate.
- Advise re likely duration: about one hour
- Check if there is anyone else in the room. Confirm that the participant is happy for that person to be in room. Ask that others in the room do not interject during the interview and advise them that it may not be possible to remove their comments from the recordings, but that these comments will not be used for the research. Be prepared to comment during the interview if there are too many interruptions.
- Explain the format of the interview – ie
    - participant invited to tell their story.
    - Define the scope – from time of onset of illness to treatment initiation.
    - "Once you have finished telling your story, I might ask you some questions."
- Advise the participant that recording will begin and turn recording devices on.
- State
    - "This is the audio recorded consent for the OAT Study".
    - For "PARTICPANT NAME" and "DATE OF BIRTH".
    - Recorded on "DATE" by "INVESTIGATOR'S NAME".
    - "I am going to read each statement from Consent Form Two. If you agree with the statement, please say "I agree". You must agree with all the statements to be eligible to take part in the study. Your consent will be audio recorded and stored securely."
    - Read each statement from Consent Form Two. Ensure that participant's agreement to each statement is clearly heard.

Kidy F, *et al. BMJ Open* 2023; 13:e070933. doi: 10.1136/bmjopen-2022-070933

Step Two: Initiation of the interview and main narration

- Advise participant that interview will begin
- "I am interested in trying to understand your experience of being diagnosed with TB. Can you tell me what happened and how you felt from the time you first became unwell until you started treatment for TB?"
- Take notes and use verbal and non-verbal prompts.
- When the narrative reaches a natural end, ask, "Is there something else you want to tell me?"

Step Three: Questioning Phase.

Questions should be formulated using the participant's own words. The questions below are a guide only.

Questions should not start with "Why" and should not be used to point out contradictions in the narrative.

Points which should be probed if they have not been covered in the narrative. Every point will not be relevant to all participants:

- Generally applicable question structure, "You mentioned XXX, can you say a bit more about that?"
- Is symptom onset clearly identified. For example, "How were you feeling before "FIRST SYMPTOMS" started?"
- Appraisal process. For example, "What made you realise that you were not well?"
- Symptoms before presenting to health care. For example, "How were you feeling before you went to see the doctor or nurse?"
- Trigger to seek health care. For example, "What made you decide to go and see the doctor or nurse?"
- Influence of others on the decision to seek health care. For example, "Did friends or family encourage or discourage you from seeing a doctor or nurse?"
- Ease of access to health care. For example, "Was it easy to get an appointment to see a doctor or nurse?"
- Experience of first and subsequent contacts with health care. For example, "What happened when you first saw a doctor or nurse?" and/ or, "How did you feel after you went to see the doctor or nurse?"
- Experience of waiting for a diagnosis. For example, "How did you feel while you were waiting for a diagnosis?"
- Experience of getting a diagnosis. For example, "What did it feel like when you found out you have TB?"
- Experience of receiving treatment. "Can you tell me more about the experience of receiving treatment?"
- Impact of COVID. "How did COVID affect you and your care?"

Personal details – if not captured in the interview

- Employment status
- Housing situation
- Country of birth

Kidy F, *et al. BMJ Open* 2023; 13:e070933. doi: 10.1136/bmjopen-2022-070933

Step Four: Concluding talk

- Ask participant if they are happy with the information shared
- Advise the participant that the recording is ending.
- Note additional information received after the recording is completed as contextual information in field notes. This will not be included in the analysis.

Kidy F, *et al. BMJ Open* 2023; 13:e070933. doi: 10.1136/bmjopen-2022-070933
