# Supplementary material for: From symptom onset to treatment initiation: protocol for a narrative study exploring the journey of older adults with tuberculosis in the English Midlands, UK
Source: BMJ Open. 2023 Nov 17;13(11):e070933. doi: 10.1136/bmjopen-2022-070933 (PMC10660672; doi:10.1136/bmjopen-2022-070933)
Supplement: Author's manuscript [file bmjopen-2022-070933.draft_revisions.pdf]

BMJ Open is committed to open peer review. As part of this commitment we make the peer review history of every article we publish publicly available.

When an article is published we post the peer reviewers' comments and the authors' responses online. We also post the versions of the paper that were used during peer review. These are the versions that the peer review comments apply to.

The versions of the paper that follow are the versions that were submitted during the peer review process. They are not the versions of record or the final published versions. They should not be cited or distributed as the published version of this manuscript.

BMJ Open is an open access journal and the full, final, typeset and author-corrected version of record of the manuscript is available on our site with no access controls, subscription charges or pay-per-view fees (http://bmjopen.bmj.com).

If you have any questions on BMJ Open's open peer review process please email info.bmjopen@bmj.com

BMJ Open

From symptom onset to treatment initiation: protocol for a narrative study exploring the journey of older adults with tuberculosis in the English Midlands, UK

Journal:	*BMJ Open*
Manuscript ID	bmjopen-2022-070933
Article Type:	Protocol
Date Submitted by the Author:	08-Dec-2022
Complete List of Authors:	Kidy, Farah; University of Warwick Warwick Medical School<br>McCarthy, Noel; Trinity College Dublin<br>Seers, Kate; University of Warwick Warwick Medical School, RCN RI
Keywords:	Tuberculosis < INFECTIOUS DISEASES, Quality in health care < HEALTH SERVICES ADMINISTRATION & MANAGEMENT, Public health < INFECTIOUS DISEASES, PRIMARY CARE

SCHOLARONE™
Manuscripts

From symptom onset to treatment initiation: protocol for a narrative study exploring the journey of older adults with tuberculosis in the English Midlands, UK

Farah Kidy[1]

Noel McCarthy[2]

Kate Seers[1]

Corresponding author:

Farah Kidy, f.kidy@warwick.ac.uk

[1] Warwick Medical School, University of Warwick, Gibbet Hill Road, Coventry, UK

[2] School of Medicine, Trinity College Dublin, College Green, Dublin 2, Ireland

For peer review only

Word count, excluding title page, abstract, references, figures and tables: 3790

KEY WORDS: Tuberculosis, treatment delay, older adults, narrative inquiry, case note review

ABSTRACT

Introduction

Time from symptom onset to treatment initiation in tuberculosis (TB) remains stubbornly prolonged despite reductions in disease incidence. Delays may contribute to increased morbidity, mortality, onward spread of disease and poor patient experiences. Most delays occur prior to hospital referral. The average primary care health care provider in England is unlikely to see TB on a regular basis. Little is known about primary care diagnostic and referral challenges.

Adults aged 65 years or older are more likely to experience delays. However, little is known about their journey from symptom onset to treatment initiation.

Methods and analysis

We will carry out a narrative study including adults aged 65 years or older, living in the English Midlands and receiving treatment for active TB. Twelve English and 12 Urdu or Punjabi speakers will be recruited from TB clinics and interviewed. Their primary care records will be accessed, and the primary care story and secondary care letters will be extracted. Each of the data sources will be analysed using dialogical narrative analysis. Data will be triangulated within participants and across the data set.

Ethics and dissemination

This study received approval from the Health Research Authority and the Research Ethics Committee in April 2022. Risk management and equity considerations have been made a priority. Findings will be disseminated through publication in open access peer reviewed journals, presentations to policy makers and through public facing materials developed in conjunction with patients, members of the pubic, TB services and charities.

ARTICLE SUMMARY

Strengths and limitations of this study

- This study takes a broad approach to exploring the pre-hospital experiences and contexts of older adults with tuberculosis.
- Using narrative methods allows for an understanding of the sequence and experience of events and opens the door to exploring causal mechanisms.
- This study gives voice to a group whose experiences are seldom considered and who are most likely to suffer from negative consequences of tuberculosis.
- Urdu and Punjabi speakers are being included alongside English speakers. However, other language groups are excluded
- Case notes provide an insight into events as they unfold but are not always purely the view of a single individual. This brings challenges to analysis and interpretation.

INTRODUCTION

Despite year-on-year decreases in the incidence of tuberculosis (TB) in England, time from symptom onset to treatment initiation remains stubbornly prolonged. Neither the proportion of people waiting (32.4% waited for 4 months or longer in 2020), nor the mean delay (median 79 days between symptom onset and treatment initiation, inter-quartile range 39-

For peer review only

154 days) have improved in the last five years.[1] For clarity, in this paper the term “treatment delay” will be used to refer to the time from symptom onset to treatment initiation regardless of the duration.

The TB Action Plan for England sets out the priorities and actions for TB services, the UK Health Security Agency (UKHSA), the NHS in England and other stakeholders to ensure that the country meets its commitment to eliminate TB as a public health problem by 2035.[2] Recovery from Covid and prevention, detection and control of TB are prioritised. Addressing treatment delays underpins activities within all these areas.

Treatment delays are a particular focus for action because they may lead to harmful consequences including increased morbidity,[3-5] mortality,[3] transmission,[3, 6, 7] and poor patient experiences [8, 9]. Since it is unclear when these effects emerge, there is no agreed qualitative or quantitative definition for how long is too long between symptom onset and treatment initiation.

For peer review only

Adults aged 65 years or older are disproportionately affected by treatment delays when they have disease in the lungs.[1] They are also more likely to develop extra-pulmonary disease which is associated with even longer delays (median 9 months in orthopaedic disease).[8, 10, 11]

Research to reduce treatment delays is most advanced in cancer studies. Here a decade of experience highlights the importance of exploring the whole patient journey. From the process of self-appraisal which identifies that there has been a shift from the norm, to the decision to seek health care and subsequent actions of health care professionals.[12] A modified version of Walter *et al*’s, 2012, model has been developed for this project, Fig 1. This highlights the importance of taking a multi-faceted view to exploring the patient journey and provides a framework to reflect on as the study progresses.

Figure 1: Showing events, processes and intervals of delay in the patient journey from symptom onset to treatment initiation.

The patient phase of delay

There is a body work exploring health care seeking behaviours in adults with TB in low incidence countries. Perceived stigma and fear of being isolated from loved ones were barriers to accessing care,[9, 13, 14] particularly amongst migrant populations.[9, 13, 15, 16] In some cases, mild symptoms were misinterpreted as being due to something which would resolve itself or could be treated at home in the context of low perceived personal risk of TB.[9, 17-20] In some settings, practical considerations such as distance to health care facility, having to pay for transport or anticipated wait times influenced the decision to seek care.[13, 15, 17] Being a new migrant or facing language barriers also delayed presentation to health care services.[19, 21] Unfortunately, few participants aged 65 or older were included in these studies.

There is a long-standing argument that older adults experience ageism at individual, policy and system levels when engaging with health care.[22] Reflecting on the patient journey, the evidence about the self-appraisal process and the decision to seek health care in older adults is mixed. In a review of studies exploring the cancer journey, age was found to be associated with both increased and decreased patient phase delays.[23] The appraisal process was influenced by symptom awareness and interpretation.[23, 24] The decision to seek health care was influenced by the support of others, independence, awareness of systems, fear of potential diagnoses and self-management including alternative medicine.[23, 24] These influences could act to both prompt or delay seeking health care. These reviews did not include participants with TB.

The healthcare phase of delay

Studies from Europe and the UK have shown greater relative contribution of either patient[25, 26] or health care[19, 27-30] stages of treatment delay. Most studies did not differentiate between the stages of health care delay, but Roberts et al, 2020, report that in the Southeast of England, most health care delay occurred prior to referral to secondary care.[21]

The knowledge gaps

To influence patient journeys, we need to understand both what happened and why it happened. This knowledge is missing for older adults with TB from patient, primary care and secondary care perspectives. The objective of this study is to address these knowledge gaps by exploring:

1. The sequence of events leading from a point of no new illness to the point of treatment initiation.
2. The experience of living through these events.
3. The impact of others (e.g., family and health care providers) on the sequence and experience of these events
4. Recognition in and referral from primary care
5. Recognition and diagnosis in secondary care

METHODS AND ANALYSIS

Ontology and epistemology

This study forms part of a larger, mixed methods research project exploring treatment delays in TB, its causes, and its consequences. Critical Realism offers a philosophical stance within which each workstream can be carried out and then integrated. Bhaskar, the founder of Critical Realism, offers a three-level description of reality. The *empirical* level is that which is observable, the *actual* level is the reality which exists irrespective of our observations and about which we may or may not be aware and lastly, the *real* level which encompasses unseen causal influences.[31] We assume that there is an *actual* patient journey from symptom onset to treatment initiation, but that our description and understanding of this will vary depending on the method of inquiry used and to whom this is delivered. Explicit acknowledgement of the *real* level allows us to delve into questions about causal mechanisms and how or when they are triggered within complex systems.[32]

For peer review only

Methodology

This study will use narrative inquiry applied to both interviews and written records. Narrative inquiry involves collecting data in the form of narratives (or stories) and considers the whole narrative as the unit of analysis. A narrative is characterized by having a beginning followed by a sequence of unfolding events, culminating with an end.[33] There is a plot and there are characters other than the narrator.[34] Narratives tell us how humans experience and make sense of the world.[35] Listening to the whole story allows us insight not only into what has happened but also why it happened and what that felt like. Compared to other qualitative methods, there is an explicit focus on understanding how the illness fits into patients' lives.[36] Narrative approaches have been used successfully to explore health care experiences of older adults[37] including those with a different first language to the investigators.[38]

Narrative inquiry fits well within the Critical Realism philosophy as there is an assumption of an *actual* patient journey with people, structures and events. We are *empirically* describing this from both participants' memories and their health records. We will use the content and structure of the narratives to come to conclusions about the *real* mechanisms underlying healthcare seeking behaviour and arrival at diagnoses.

Sample size

Determining sample size for qualitative research is complex. Some researchers believe that *a priori* determination of sample sizes is not possible[39] rather that data saturation or data adequacy be used.[40] Others suggest that for non-probabilistic sampling in homogenous populations, most themes are apparent after six interviews and that saturation is reached after 12 interviews.[41] However, Hennick et al, 2017, distinguish between code saturation (heard it all) and meaning saturation (understood it all).[42] Specifically, within narrative methods, numbers can vary from individual case studies[43] to as many as forty participants.[44] With the aim of ensuring meaning saturation of the main narrative themes, we will recruit up to 24 patients, with up to twelve who speak English and up to twelve who speak Urdu or Punjabi.

Recruitment and Eligibility

Participants will be identified from TB clinics in the English Midlands by members of their normal care team.

Patients will be considered eligible if they satisfy all the following criteria:

- aged 65 years or older at the time of diagnosis
- any gender
- resident in the catchment area
- English, Urdu or Punjabi speakers.
- receiving treatment for active TB
- managed in the community
- able to give informed consent
- hear well enough to have a remote conversation.

Urdu and Punjabi were selected from amongst other languages as they are the most frequently spoken non-English languages in our catchment area.

For peer review only

A stratified purposive sampling approach will be taken. Participants will first be stratified by language (English or Urdu/Punjabi speaking) and then gender (See Fig 2.) Within each stratum, there will be purposive sampling to select those with differing sites of disease (pulmonary or extra-pulmonary), a range of delay times and a spectrum of ages over 65 years. To avoid bias, outstanding strata for recruitment will be regularly reviewed with the clinical teams.

Figure 2: Showing planned stratification of participants

Participants will be given the option of taking part in the interview with or without the case note review.

Participants will be offered a £20 voucher for taking part in the study.

For peer review only

Data Collection

Data will be collected from the participant and from their primary care record generating a patient story, a primary care story as recorded in the notes and a secondary care story as recorded in letters.

The interview

Participant experience will be captured using a narrative interview. The narrative interview is an unstructured, in-depth interview in which the influence of the interviewer is minimized.[45] A modification of the schema suggested by Jovechelovitch and Bauer, 2000, will be applied.[45]

1. Initiation of the interview. An open question will be asked which defines the story of interest, its start point and its end point.
2. Main narration. During this phase there will be minimal interruptions, with simple verbal and non-verbal cues to encourage the participant.
3. Questioning phase. Key events of interest will be identified prior to the interview and the participant's own words will be used to probe further about these events if they have not already been discussed. However, this will not include seeking opinions or judgements.
4. Concluding talk. During this phase, member checking will be carried out by directly asking participants if they are happy with the information shared.

A topic guide has been developed to ensure that key turning points in the story are captured (Supplementary material 1). This will be tested with one English speaking and one Urdu or Punjabi speaking participant and adapted as needed.

Interviews will be transcribed verbatim in the language of the participant and translated into English where needed. Data will be managed by using NVivo.

Primary Care Records

The eligibility time for extraction from records will start from twelve months prior to the date of symptom onset as reported by participants and will end at the date of treatment initiation

as reported by the TB clinic. This long look-back period is chosen since there is limited evidence on the likely errors in recall of onset date.[46]

A list of alert symptoms for TB has been developed for another part of this project. This includes general symptoms such as fever, night sweats and weight loss; site-specific symptoms such as productive cough or enlarged lymph nodes for disease in the lungs or lymph nodes, respectively; and tests commonly carried out in primary care which may be abnormal in TB. Any consultation, within the eligible time, in which these signs, symptoms, or TB disease manifestations uncovered during interviews are mentioned will be extracted. Where a consultation deals with more than one problem, and the TB related entry can be clearly differentiated, then only this entry will be extracted. A frequency count of tests will be noted, but only abnormal tests results will be extracted. Letters to and from secondary care within the eligible time frame which are related to the TB journey will also be extracted.

Relevant consultations will be identified by checking the case notes from the beginning of the eligibility period. This approach was selected as using search functions may miss data where there are misspellings or unusual abbreviations. Date of first presentation, date of referral, frequency of consultations in the eligible time, number of team members consulted, number of secondary care clinics attended, and presence of co-morbidities will be recorded and used to describe participants.

Cross cultural research

There is co-creation of the narrative between the researcher and the narrator; the story told is influenced by the audience.[47] In this study, for 12 of the participants, interpreters will be gathering data with minimal interruptions to translate the content. To aid this process, interpreters will be recruited from amongst bilingual members of the university communities who have research exposure or experience before resorting to use of formal translation services. Interpreters will be given training to cover the rationale and methods used in this project.

Stories gathered in Urdu or Punjabi will be analysed in English. In order to retain the depth of detail and nuance of the data, we will apply an approach suggested by Lopez *et al*.[48] Where the interview was conducted by an interpreter, the audio recording will be translated by an independent person with the aim of conceptual equivalence. The second translator will be asked to highlight where idioms have been used and what these mean.[49] A university-based interpreter will be asked to review the written translations and compare these to the audio recordings. Any discrepancies will be resolved by discussion with the study team.

Data Analysis

Narrative analysis refers to a collection of different methods with the shared characteristic of analysing narratives as a whole unit. This group of methods was chosen since they allow investigation of events, the experience of living through those events, the socio-cultural contexts of the participants and the way in which the participant has chosen to tell their story. These features are important in achieving the objectives of this study.

The three data sources (interview, primary care record and secondary care letters) for each patient are distinct stories with different narrators and viewpoints. The main analysis will therefore treat each data source separately. Additionally, based on the Critical Realism mindset which sees each data source as a different tool to capture the *actual* patient journey, we will explore the data through triangulation after matching data sources for individual patients.

For peer review only

We will carry out dialogical narrative analysis using a method recommended by Smith, 2016.[34] Although this is presented as a numbered list, the process is iterative, and steps two to five will be repeated several times:

1. “Indwelling” – this is the process of familiarization by reading the transcript several times.

2. Identifying or smoothing the story – at this stage the “core story” or stories are identified. This enables shortening of the narrative to aid further analysis.

   For the interviews, comments from the interviewer will be removed. The text will be re-arranged to provide a chronological arc to the story. Extraneous material (if any) will be marked in a different colour.[50]

   For the textual data sources portions of the consultations or letters which refer to health problems which are clearly different to TB will be removed. Again, data will be arranged chronologically if needed.

3. Identifying narrative themes – rather than line by line coding, the aim here is to identify themes which run through the whole story. At this stage we are most interested in exploring what is said. Both manifest and latent themes may be present.

4. Identifying the structure of stories – the aim here is to understand “how the story is put together”.[34] At this stage we are interested in identifying how things have changed over time. For example, there may be stories of progress or stability over time.

5. Opening up the analysis – at this stage Smith recommends asking a series of questions of the stories.
   a. Resource questions. Why is the person telling this story in this way? What templates of narratives are they drawing on to tell this story?
   b. Circulation questions. Who is the intended audience?
   c. Connection questions. Does this story create a “them” and “us”? Is there someone the storyteller does not want to hear this story?
   d. Identity questions. Does the story give the person a sense of identity? Or does it identify who they might want to be in the future?
   e. Function questions. Does the story help or hinder the storyteller? What effect can or could the story have on other people?

Twenty percent of the data will also be analysed by KS to ensure that no core stories or narrative themes are missed.

The results will be drawn together by building a typology of stories. Stories from within each data source and across the whole data set will be clustered together according to shared characteristics in the domains of themes, structure, resources and function.

Triangulation

The approach to triangulation will follow a method suggested by Farmer *et al*, 2006.[51] Data will be triangulated within participants and across the whole data set by looking for convergence, completeness, silence and dissonance across the domains of narrative themes, story structure, resources and function. This will allow us to understand how the perspectives of the story tellers impact on the story told.

For peer review only

Bringing rigour

Trustworthiness and plausibility[52] will be evidenced through member checking at the end of the interview by asking participants if they are happy with all the material they have shared. The context of interviews and an explanation of data gathering techniques will also be provided in the report.

In order to increase criticality,[52] FK will keep a contemporaneous reflexive diary to help understand her role as a researcher rather than GP, and to explore her own biases and preconceptions.

ETHICS AND DISSEMINATION

Ethics

Some of the key ethical issues raised by the study are discussed below.

Equity

TB often affects the most vulnerable in society and is seen in those with chaotic lifestyles. It is important that we include these voices in our research. TB teams have been sensitised to identification of participants based on the eligibility criteria alone. If there are concerns about violence towards staff or difficulty engaging, participants will be offered remote interviews only.

TB affects migrant populations. Our approach to recruitment means that English speaking migrants from a range of backgrounds can be included. However, we have restricted the number of languages included and this means that some voices will not be heard.

We have made the decision to transcribe all interviews in the language of the participants to allow reporting in the source language and to ensure that all participants' original words are available for secondary analysis.

Recruitment

The duration of TB treatment means that clinical teams and patients may develop strong relationships. To reduce the risk of participants feeling obliged to participate, they will be made aware that non-participation will not negatively affect their care and that they are free to withdraw at any time. Although the clinical teams will identify potential participants, consent will be obtained by the research team.

Harms to participants

Some participants may find it distressing to talk about their experiences. Should this occur, the interview will be paused or terminated. FK is an experienced clinician and has training and experience in de-escalating situations. The nature of narrative interviews means that participants are in control of the level of detail they reveal. Participants will be allowed time to debrief and will be signposted to local support services if required.

Interviews will take place remotely or in clinical spaces and during normal working hours to limit potential environmental harms to both participants and researchers.

Confidentiality

For peer review only

Data will be stored in anonymised, encrypted, electronic formats. IT Security Teams have been consulted to ensure that data storage and transfer procedures are appropriate. Aspects of the narrative which might lead to identification of participants will not be reported.

The patient information leaflet clarifies that there may be situations in which disclosures are needed. In line with General Medical Council, UK, guidelines,[53] participants will first be asked for permission to disclose information. If permission is not forthcoming, then identifiable information will only be shared if this is in the interests of the patient or the public. All disclosures will be noted as part of the participants research record.

Infection control concerns
Face to face interviews will only take place after the TB team confirm that this is safe. The use of personal protective equipment and distancing measures will be determined by national guidelines, University and NHS Trust policies.

Case note reviews
Accessing case notes may make participants feel exposed or vulnerable. To this end, participants will be able to consent to the interview study alone.

Data extraction will be kept proportionate by using a pre-identified list of symptoms and signs or the participant's own words to identify appropriate material for the study.

Case note review may also make participants' GPs feel exposed. A graded approach will be taken to handling potential risks to participant safety. Where there is an ongoing or active risk, for example monitoring blood tests not being carried out, this will be flagged up using an auditable electronic messaging service.

Where there is the suggestion of historic risk to patient safety, for example avoidable treatment delay, the surgery will be made aware of this as a potential significant event. Preferred routes and recipients of each type of communication will be determined on a surgery-by-surgery basis.

This study has been approved by the National Health Service Health Research Authority and the London and Harrow Ethics Committee in April 2022 (ref: 22/PR/0357).

Dissemination
This study forms part of a larger body work which will be submitted as a PhD thesis. The full thesis will be published on the University of Warwick website. Following analysis, a plain language summary will be shared with participants. Findings will be shared in peer reviewed journals and through public facing materials developed in conjunction with TB services, primary care colleagues, patient partners and TB charities.

Typologies generated by this study will be used to highlight events, mechanisms and trajectories which should raise suspicion of TB.

PPI STATEMENT

People with living TB, older adults with chronic conditions and their carers were consulted about the project aims, methods and implementation. This directly influenced the decision to

For peer review only

interview participants alone, impacted on the locations for interviews and the methods of dissemination. Patient partners commented on the information leaflets and consent forms. Bilingual members of the public helped to recruit translators.

FUNDING STATEMENT

FK is funded by the NIHR Doctoral Research Fellowship, grant number 300688. The views expressed are those of the author(s) and not necessarily those of the NIHR or the Department of Health and Social Care.

The project is being delivered with support from the Clinical Research Networks for Primary and Secondary Care in the West Midlands.

COMPETING INTERESTS STATEMENT

There are no competing interests to declare.

AUTHOR STATEMENT

FK conceived the study with support from KS and NMc

FK wrote the first draft of the paper and all authors commented on revisions.

DATA STATEMENT SECTION

Interview transcripts will be stored for ten years after the end of the study and will made available for secondary analysis on a case-by-case basis.

REFERENCES

1. UK Health Security Agency, *Tuberculosis in England. 2021 report (Presenting data to end 2020)*. 2021, UKHSA: UK.
2. Agency, U.H.S., *TB Action Plan for England, 2021 to 2026*. 2021: London.
3. Greenaway, C., et al., *Delay in Diagnosis among Hospitalized Patients with Active Tuberculosis—Predictors and Outcomes.* American Journal of Respiratory and Critical Care Medicine, 2002. **165**(7): p. 927-933.
4. Virenfeldt, J., et al., *Treatment delay affects clinical severity of tuberculosis: a longitudinal cohort study.* BMJ Open, 2014. **4**.
5. Cheng, S., et al., *Effect of Diagnostic and Treatment Delay on the Risk of Tuberculosis Transmission in Shenzhen, China: An Observational Cohort Study, 1993–2010.* PLOS ONE, 2013. **8**(6): p. e67516.
6. Golub, J.E., et al., *Delayed tuberculosis diagnosis and tuberculosis transmission.* The International Journal of Tuberculosis and Lung Disease, 2006. **10**(1): p. 24-30.
7. Melsew, Y.A., et al., *Risk factors for infectiousness of patients with tuberculosis: a systematic review and meta-analysis.* Epidemiology and Infection, 2018. **146**(3): p. 345-353.
8. NICE. *NICE Guideline 33 - Tuberculosis*. 2016 27.08.2018]; Available from: https://www.nice.org.uk/guidance/ng33.
9. Nnoaham, K.E., et al., *Perceptions and experiences of tuberculosis among African patients attending a tuberculosis clinic in London.* The International Journal of Tuberculosis and Lung Disease, 2006. **10**(9): p. 1013-1017.
10. Broderick, C., et al., *Delays in the diagnosis and treatment of bone and joint tuberculosis in the United Kingdom.* The Bone & Joint Journal, 2018. **100-B**(1): p. 119-124.

For peer review only

11. Abbara, A., et al., *Time to diagnosis of tuberculosis is greater in older patients: a retrospective cohort review.* ERJ open research, 2019. **5**(4): p. 00228-2018.
12. Walter, F., et al., *The Andersen Model of Total Patient Delay: a systematic review of its application in cancer diagnosis.* Journal of health services research & policy, 2012. **17**(2): p. 110-118.
13. Quattrocchi, A., et al., *Determinants of patient and health system delay among Italian and foreign-born patients with pulmonary tuberculosis: a multicentre cross-sectional study.* BMJ Open, 2018. **8**.
14. Wieland, M.L., et al., *Perceptions of tuberculosis among immigrants and refugees at an adult education center: a community-based participatory research approach.* Journal of immigrant and minority health, 2012. **14**(1): p. 14-22.
15. Abarca Tomás, B., et al., *Tuberculosis in Migrant Populations. A Systematic Review of the Qualitative Literature.* PLOS ONE, 2013. **8**(12): p. e82440.
16. Gerrish, K., A. Naisby, and M. Ismail, *The meaning and consequences of tuberculosis among Somali people in the United Kingdom.* Journal of Advanced Nursing, 2012. **68**(12): p. 2654-2663.
17. Asch, S., et al., *Why Do Symptomatic Patients Delay Obtaining Care for Tuberculosis?* American Journal of Respiratory and Critical Care Medicine, 1998. **157**(4): p. 1244-1248.
18. Calder, L., W. Gao, and G. Simmons, *Tuberculosis: reasons for diagnostic delay in Auckland.* N Z Med J, 2000. **113**: p. 483.
19. Peri, A.M., et al., *Determinants of patient and health care services delays for tuberculosis diagnosis in Italy: a cross-sectional observational study.* BMC Infectious Diseases, 2018. **18**(1): p. 690.
20. Craig, G.M., A. Joly Lm Fau - Zumla, and A. Zumla, *'Complex' but coping: experience of symptoms of tuberculosis and health care seeking behaviours--a qualitative interview study of urban risk groups, London, UK.* BMC Public Health, (1471-2458 (Electronic)).
21. Roberts, D.J., et al., *Factors associated with delay in treatment initiation for pulmonary tuberculosis.* ERJ Open Research, 2020. **6**.
22. Wyman, M.F., S. Shiovitz-Ezra, and J. Bengel, *Ageism in the Health Care System: Providers, Patients, and Systems*, in *Contemporary Perspectives on Ageism*, L. Ayalon and C. Tesch-Römer, Editors. 2018, Springer International Publishing: Cham. p. 193-212.
23. Jones, D., et al., *Factors influencing symptom appraisal and help-seeking of older adults with possible cancer: a mixed-methods systematic review.* British Journal of General Practice, 2022. **72**(723): p. e702.
24. Teo, K., et al., *Help-Seeking Behaviors Among Older Adults: A Scoping Review.* Journal of Applied Gerontology, 2022. **41**(5): p. 1500-1510.
25. Paynter, S., et al., *Patient and health service delays in initiating treatment for patients with pulmonary tuberculosis: retrospective cohort study.* The International Journal of Tuberculosis and Lung Disease, 2004. **8**(2): p. 180-185.
26. Loutet, M.G., et al., *Delay from symptom onset to treatment start among tuberculosis patients in England, 2012–2015.* Epidemiology and Infection, 2018. **146**(12): p. 1511-1518.
27. Tattevin, P., et al., *Factors associated with patient and health care system delay in the diagnosis of tuberculosis in France.* The International Journal of Tuberculosis and Lung Disease, 2012. **16**(4): p. 510-515.
28. Saldana, L., et al., *Factors affecting delay in initiation of treatment of tuberculosis in the Thames Valley, UK.* (1476-5616 (Electronic)).
29. Farah, M.G., et al., *Patient and health care system delays in the start of tuberculosis treatment in Norway.* BMC infectious diseases, 2006. **6**: p. 33-33.
30. Sultan, H., S. Haroon, and N. Syed, *Delay and completion of tuberculosis treatment: a cross-sectional study in the West Midlands, UK.* Journal of Public Health, 2013. **35**(1): p. 12-20.

For peer review only

31. Alderson, P., *Critical realism for health and illness research: A practical introduction*. 2021: Policy Press.
32. Koopmans, E. and D.C. Schiller, *Understanding Causation in Healthcare: An Introduction to Critical Realism.* Qualitative Health Research, 2022. **32**(8-9): p. 1207-1214.
33. Greenhalgh, T. and B. Hurwitz, *Why study narrative?* BMJ, 1999. **318**: p. 48-50.
34. Smith B, *Narrative Analysis*, in *Analysing Qualitative Data in Psychology*, Lyons E and C. A, Editors. 2016, Sage: London.
35. Connelly, F.M. and D.J. Clandinin, *Stories of Experience and Narrative Inquiry.* Educational Researcher, 1990. **19**(5): p. 2-14.
36. Burck, C., *Comparing qualitative research methodologies for systemic research: the use of grounded theory, discourse analysis and narrative analysis.* Journal of Family Therapy, 2005. **27**(3): p. 237-262.
37. Hsu, M.Y. and B. McCormack, *Using narrative inquiry with older people to inform practice and service developments.* Journal of Clinical Nursing, 2012. **21**(5-6): p. 841-849.
38. Schwind, J.K., et al., *What can be learned from patient stories about living with the chronicity of heart illness? A narrative inquiry.* Contemporary Nurse, 2016. **52**(2-3): p. 216-229.
39. Sim, J., et al., *Can sample size in qualitative research be determined a priori?* International Journal of Social Research Methodology, 2018. **21**(5): p. 619-634.
40. Vasileiou, K., et al., *Characterising and justifying sample size sufficiency in interview-based studies: systematic analysis of qualitative health research over a 15-year period.* BMC Medical Research Methodology, 2018. **18**(1): p. 148.
41. Guest, G., A. Bunce, and L. Johnson, *How Many Interviews Are Enough?:An Experiment with Data Saturation and Variability.* Field Methods, 2006. **18**(1): p. 59-82.
42. Hennink, M.M., B.N. Kaiser, and V.C. Marconi, *Code Saturation Versus Meaning Saturation:How Many Interviews Are Enough?* Qualitative Health Research, 2017. **27**(4): p. 591-608.
43. Vlase, I. and R. Sieber, *Narrating well-being in the context of precarious prosperity: An account of agency framed by culturally embedded happiness and gender beliefs.* European Journal of Women's Studies, 2016. **23**(2): p. 185-199.
44. Mu'Min Chowdhury, A., C. Helman, and T. Greenhalgh, *Food beliefs and practices among British Bangladeshis with diabetes: Implications for health education.* Anthropology & Medicine, 2000. **7**(2): p. 209-226.
45. Jovchelovitch, S. and M.W. Bauer. *Narrative interviewing {online}*. originally published in Bauer, Martin W.; Gaskell, G.; Eds., Qualitative researching with text, image and sound : a practical handbook. London, England: SAGE Publications, 2000. 2000 15.05.2020]; Available from: http://eprints.lse.ac.uk/2633
46. Schmier, J.K. and M.T. Halpern, *Patient recall and recall bias of health state and health status.* Expert Review of Pharmacoeconomics & Outcomes Research, 2004. **4**(2): p. 159-163.
47. Haydon, G. and P. van der Riet, *Narrative inquiry: A relational research methodology suitable to explore narratives of health and illness.* Nordic Journal of Nursing Research, 2016. **37**(2): p. 85-89.
48. Lopez, G.I., et al., *Translation Barriers in Conducting Qualitative Research With Spanish Speakers.* Qualitative Health Research, 2008. **18**(12): p. 1729-1737.
49. Wong, J.P.-H. and M.K.-L. Poon, *Bringing Translation Out of the Shadows: Translation as an Issue of Methodological Significance in Cross-Cultural Qualitative Research.* Journal of Transcultural Nursing, 2010. **21**(2): p. 151-158.
50. Emden, C., *Conducting a narrative analysis.* Collegian, 1998(1322-7696 (Print)).
51. Farmer, T., et al., *Developing and Implementing a Triangulation Protocol for Qualitative Health Research.* Qualitative Health Research, 2006. **16**(3): p. 377-394.

For peer review only

52. Greenhalgh, T., *Cultural contexts of health: the use of narrative research in the health sector*, in *Health Evidence Network (HEN) synthesis report*. 2016, WHO Regional Office for Europe: Copenhagen.

53. Council, G.M. *Ethical and legal duties of confidentiality*. 2020 30.07.2020]; Available from: https://www.gmc-uk.org/ethical-guidance/ethical-guidance-for-doctors/confidentiality/disclosures-for-the-protection-of-patients-and-others.

For peer review only

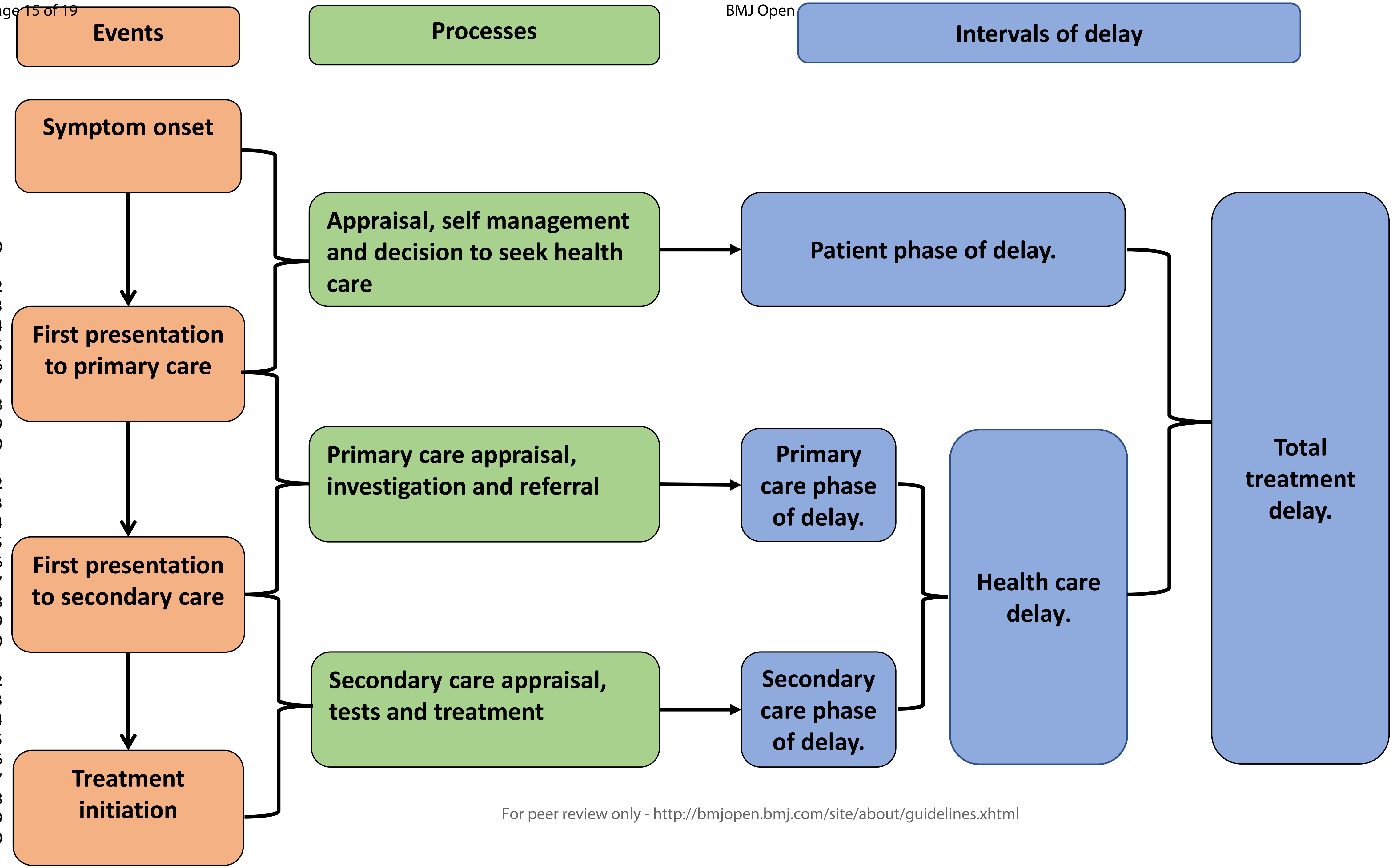

Events
Processes
Intervals of delay
Symptom onset
Appraisal, self management and decision to seek health care
Patient phase of delay.
First presentation to primary care
Primary care appraisal, investigation and referral
Primary care phase of delay.
Total treatment delay.
Health care delay.
First presentation to secondary care
Secondary care appraisal, tests and treatment
Secondary care phase of delay.
Treatment initiation

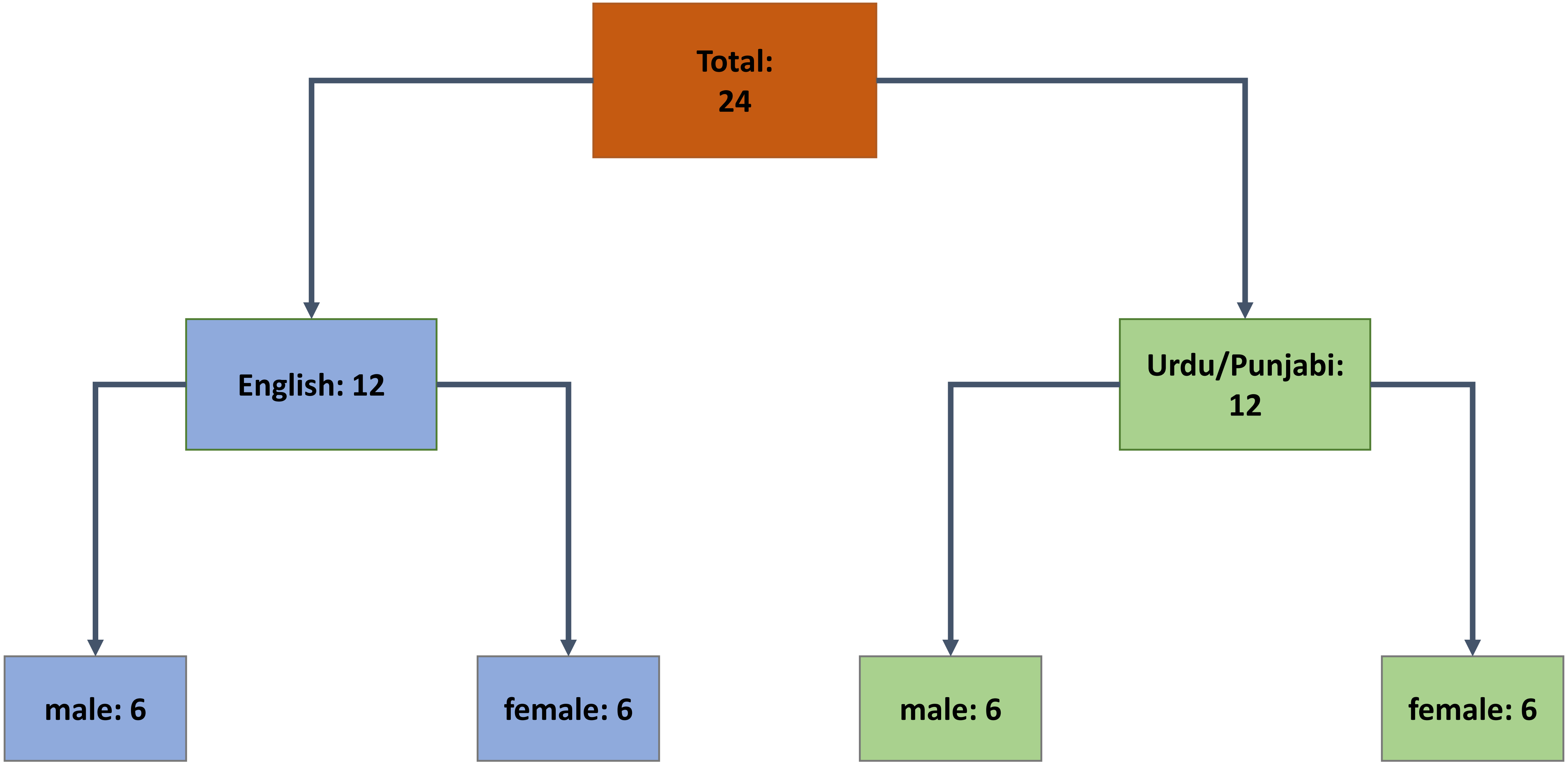
Total:
24
English: 12
Urdu/Punjabi:
12
male: 6
female: 6
male: 6
female: 6

Supplementary Material

From symptom onset to treatment initiation: A narrative study of Older Adults with Tuberculosis (OAT)

The OAT Study

Topic Guide

(Date 20/09/2020 Version 1.0)

For peer review only

The OAT Study will take a narrative approach to data gathering. The guide will the trialled on English and non-English speaking participants and adapted if needed.

Step One: Introductions, consent and identity confirmation

- Introduce self and confirm that the call is to take consent and carry out the interview.
- Ask participant to confirm their name and date of birth.
- Confirm that this is a suitable time.
- Ask participant if they have any questions about the information sheets, consent form or study in general.
- Thanks for agreeing to participate.
- Advise re likely duration: about one hour
- Check if there is anyone else in the room. Confirm that the participant is happy for that person to be in room. Ask that others in the room do not interject during the interview and advise them that it may not be possible to remove their comments from the recordings, but that these comments will not be used for the research. Be prepared to comment during the interview if there are too many interruptions.
- Explain the format of the interview – ie
    - participant invited to tell their story.
    - Define the scope – from time of onset of illness to treatment initiation.
    - "Once you have finished telling your story, I might ask you some questions."
- Advise the participant that recording will begin and turn recording devices on.
- State
    - "This is the audio recorded consent for the OAT Study".
    - For "PARTICPANT NAME" and "DATE OF BIRTH".
    - Recorded on "DATE" by "INVESTIGATOR'S NAME".
    - "I am going to read each statement from Consent Form Two. If you agree with the statement, please say "I agree". You must agree with all the statements to be eligible to take part in the study. Your consent will be audio recorded and stored securely."
    - Read each statement from Consent Form Two. Ensure that participant's agreement to each statement is clearly heard.

For peer review only - http://bmjopen.bmj.com/site/about/guidelines.xhtml

Step Two: Initiation of the interview and main narration

- Advise participant that interview will begin
- "I am interested in trying to understand your experience of being diagnosed with TB. Can you tell me what happened and how you felt from the time you first became unwell until you started treatment for TB?"
- Take notes and use verbal and non-verbal prompts.
- When the narrative reaches a natural end, ask, "Is there something else you want to tell me?"

Step Three: Questioning Phase.

Questions should be formulated using the participant's own words. The questions below are a guide only.

Questions should not start with "Why" and should not be used to point out contradictions in the narrative.

Points which should be probed if they have not been covered in the narrative. Every point will not be relevant to all participants:

- Generally applicable question structure, "You mentioned XXX, can you say a bit more about that?"
- Is symptom onset clearly identified. For example, "How were you feeling before "FIRST SYMPTOMS" started?"
- Appraisal process. For example, "What made you realise that you were not well?"
- Symptoms before presenting to health care. For example, "How were you feeling before you went to see the doctor or nurse?"
- Trigger to seek health care. For example, "What made you decide to go and see the doctor or nurse?"
- Influence of others on the decision to seek health care. For example, "Did friends or family encourage or discourage you from seeing a doctor or nurse?"
- Ease of access to health care. For example, "Was it easy to get an appointment to see a doctor or nurse?"
- Experience of first and subsequent contacts with health care. For example, "What happened when you first saw a doctor or nurse?" and/ or, "How did you feel after you went to see the doctor or nurse?"
- Experience of waiting for a diagnosis. For example, "How did you feel while you were waiting for a diagnosis?"
- Experience of getting a diagnosis. For example, "What did it feel like when you found out you have TB?"
- Experience of receiving treatment. "Can you tell me more about the experience of receiving treatment?"
- Impact of COVID. "How did COVID affect you and your care?"

Step Four: Concluding talk

- Ask participant if they are happy with the information shared
- Advise the participant that the recording is ending.

For peer review only - http://bmjopen.bmj.com/site/about/guidelines.xhtml

For peer review only

- Note additional information received after the recording is completed as contextual information in field notes. This will not be included in the analysis.

For peer review only

For peer review only - http://bmjopen.bmj.com/site/about/guidelines.xhtml

BMJ Open

From symptom onset to treatment initiation: protocol for a narrative study exploring the journey of older adults with tuberculosis in the English Midlands, UK

Journal:	*BMJ Open*
Manuscript ID	bmjopen-2022-070933.R1
Article Type:	Protocol
Date Submitted by the Author:	26-Jul-2023
Complete List of Authors:	Kidy, Farah; University of Warwick Warwick Medical School<br>McCarthy, Noel; Trinity College Dublin<br>Seers, Kate; University of Warwick Warwick Medical School, RCN RI
**Primary Subject Heading**:	Public health
Secondary Subject Heading:	Epidemiology, General practice / Family practice, Infectious diseases, Qualitative research
Keywords:	Tuberculosis < INFECTIOUS DISEASES, Quality in health care < HEALTH SERVICES ADMINISTRATION & MANAGEMENT, Public health < INFECTIOUS DISEASES, PRIMARY CARE

SCHOLARONE™
Manuscripts

From symptom onset to treatment initiation: protocol for a narrative study exploring the journey of older adults with tuberculosis in the English Midlands, UK

Farah Kidy[1]

Noel McCarthy[2]

Kate Seers[1]

Corresponding author:

Farah Kidy, f.kidy@warwick.ac.uk

[1] Warwick Medical School, University of Warwick, Gibbet Hill Road, Coventry, UK

[2] School of Medicine, Trinity College Dublin, College Green, Dublin 2, Ireland

Word count, excluding title page, abstract, references, figures and tables: 3790

KEY WORDS: Tuberculosis, treatment delay, older adults, narrative inquiry, case note review

For peer review only

ABSTRACT

Introduction

Time from symptom onset to treatment initiation in tuberculosis (TB) remains stubbornly prolonged despite reductions in disease incidence. Delays may contribute to increased morbidity, mortality, onward spread of disease and poor patient experiences. Most delays occur prior to hospital referral. The average primary care health care provider in England is unlikely to see TB on a regular basis. Little is known about primary care diagnostic and referral challenges.

Adults aged 65 years or older are more likely to experience delays. However, little is known about their journey from symptom onset to treatment initiation.

Methods and analysis

We will carry out a narrative study including adults aged 65 years or older, living in the English Midlands and receiving treatment for active TB. Twelve English and 12 Urdu or Punjabi speakers will be recruited from TB clinics and interviewed. Their primary care records will be accessed, and the primary care story and secondary care letters will be extracted. Each of the data sources will be analysed using dialogical narrative analysis. Data will be triangulated within participants and across the data set.

Ethics and dissemination

This study received approval from the Health Research Authority and the Research Ethics Committee in April 2022. Risk management and equity considerations have been made a priority. Findings will be disseminated through publication in open access peer reviewed journals, presentations to policy makers, primary healthcare and secondary healthcare professionals, and through public facing materials developed in conjunction with patients, members of the pubic, TB services and charities.

ARTICLE SUMMARY

Strengths and limitations of this study

- Using narrative methods allows for an understanding of the sequence and experience of events and opens the door to exploring causal mechanisms.
- This study will recruit participants whose experiences are seldom considered and who are most likely to suffer from negative consequences of tuberculosis.
- Including only Urdu, Punjabi and English speakers is a potential limitation.
- Case notes may be challenging to analyse and interpret.

INTRODUCTION

England is a low-incidence tuberculosis (TB) country with an incidence of 7.8 per 100,000 in 2021.[1] Despite decreases in the incidence of TB since 2011, time from symptom onset to treatment initiation (treatment delay) remains stubbornly prolonged. Neither the proportion of people waiting (31.6% waited for 4 months or longer in 2021), nor the median delay (79 days, inter-quartile range 40-153) have improved in the last five years.[1]

The TB Action Plan for England sets out the priorities and actions for TB services, the UK Health Security Agency (UKHSA), the NHS in England and other stakeholders to ensure that

For peer review only

the country meets its commitment to eliminate TB as a public health problem by 2035.[2] Recovery from Covid and prevention, detection and control of TB are prioritised. Addressing treatment delays underpins activities within all these areas.

Treatment delays are a particular focus for action because they may lead to harmful consequences including increased morbidity,[3-5] mortality,[3] transmission,[3, 6, 7] and poor patient experiences [8, 9].

Adults aged 65 years or older contribute 16% of cases but make greater contribution to those who experience delays of 4 months or longer, Fig 1.[10-16] These data only show cases with pulmonary TB. Older adults are also more likely to develop extra-pulmonary disease which is associated with even longer delays (median 9 months in orthopaedic disease).[8, 17, 18] A greater proportion of this age group require additional support during treatment (37% vs 32.9% of 45 to 64 year olds, or 28.2% of 15 to 44 year olds) and they are less likely to complete treatment than their younger counterparts (65.6% vs 84.8%, or 88.3% respectively).[1]

Figure 1: Showing trends in the proportion of patients in England with pulmonary TB experiencing treatment delays of four months or longer

Research to reduce treatment delays is most advanced in cancer studies. Here a decade of experience highlights the importance of exploring the whole patient journey. From the process of self-appraisal which identifies that there has been a shift from the norm, to the decision to seek health care and subsequent actions of health care professionals.[19] A modified version of Walter *et al*'s, 2012, model provides a framework to reflect on as the study progresses, Fig 2.

Figure 2: Showing events, processes and intervals of delay in the patient journey from symptom onset to treatment initiation.

The patient phase of delay

There is a body work (using methods such as focus groups[20], interviews[9, 21, 22], and surveys or questionnaires[23-26]) exploring health care seeking behaviours in adults with TB in low incidence countries. Perceived stigma and fear of being isolated from loved ones were barriers,[9, 20, 23] particularly amongst migrant populations.[9, 21, 23, 27] In some cases, mild symptoms were misinterpreted as being due to something which would resolve itself or could be treated at home.[9, 22, 24-26] In some settings, practical considerations such as distance to health care facility, having to pay for transport or anticipated wait times were barriers.[23, 24, 27] Being a new migrant or facing language barriers delayed presentation to health care services.[26, 28] Unfortunately, few participants aged 65 or older were included in these studies.

There is a long-standing argument that older adults experience ageism at individual, policy and system levels when engaging with health care.[29] The evidence about self-appraisal and health care seeking decisons in older adults is mixed. In a review of cancer studies, age

For peer review only

was found to be associated with both increased and decreased patient phase delays.[30] The appraisal process was influenced by symptom awareness and interpretation.[30, 31] The decision to seek health care was influenced by the support of others, independence, awareness of systems, fear of potential diagnoses and self-management including alternative medicine.[30, 31] These influences could both prompt or delay seeking health care. These reviews did not include participants with TB.

The healthcare phase of delay

Studies from Europe and the UK have shown greater relative contribution of either patient[32, 33] or health care[26, 34-37] stages of treatment delay. In our context, TB is diagnosed in secondary care settings (ie outpatient specialist clinics or during an inpatient admission).Most studies did not differentiate between the stages of health care delay, but Roberts et al, 2020, report that most health care delay occurred prior to referral to secondary care.[28]

The knowledge gaps

To influence patient journeys, we need to understand both what happened and why it happened. This knowledge is missing for older adults with TB from patient, primary and secondary care perspectives. The objective of this study is to address these knowledge gaps by exploring:

1. The sequence of events leading from a point of no new illness to the point of treatment initiation.
2. The experience of living through these events.
3. The impact of others (e.g., family and health care providers) on the sequence and experience of these events
4. Recognition in and referral from primary care
5. Recognition and diagnosis in secondary care

METHODS AND ANALYSIS

Ontology and epistemology

This study forms part of a larger, mixed methods research project exploring treatment delays in TB, its causes, and its consequences. A summary of associated studies can be seen on the funder's website.[38] Critical Realism offers a philosophical stance within which each workstream can be carried out and then integrated. Bhaskar, the founder of Critical Realism, offers a three-level description of reality. The *empirical* level is that which is observable, the *actual* level is the reality which exists irrespective of our observations and about which we may or may not be aware and lastly, the *real* level which encompasses unseen causal influences.[39] We assume that there is an *actual* patient journey from symptom onset to treatment initiation, but that our description and understanding of this will vary depending on the method of inquiry used and to whom this is delivered. Explicit acknowledgement of the *real* level allows us to delve into questions about causal mechanisms and how or when they are triggered within complex systems.[40]

Methodology

For peer review only

This study will use narrative inquiry applied to interviews and written records. Data are collected in the form of narratives (or stories) and the whole narrative is the unit of analysis. A narrative has a beginning followed by a sequence of unfolding events, culminating with an end.[41] There is a plot and there are characters other than the narrator.[42] Narratives tell us how humans experience and make sense of the world.[43] Listening to the whole story allows us insight not only into what has happened but also why it happened and what that felt like. There is an explicit focus on understanding how the illness fits into patients' lives.[44] Narrative approaches have been used successfully to explore health care experiences of older adults[45] including those with a different first language to the investigators.[46]

Narrative methods will work well for our population since they allow participants to tell the story that they think is important when they are neither seeking healthcare nor are there any negative consequences to their stories.

Narrative inquiry fits well within Critical Realism as there is an assumption of an *actual* patient journey with people, structures and events. We are *empirically* describing this from both participants' memories and their health records. We will use the content and structure of the narratives to come to conclusions about the *real* mechanisms underlying healthcare seeking behaviour and arrival at diagnoses.

Sample size

Some researchers believe that *a priori* determination of sample sizes is not possible[47] rather that data saturation or data adequacy be used.[48] Others suggest that for non-probabilistic sampling in homogenous populations, most themes are apparent after six interviews and that saturation is reached after 12 interviews.[49] However, Hennick et al, 2017, distinguish between code saturation (heard it all) and meaning saturation (understood it all).[50] With the aim of ensuring meaning saturation of the main narrative themes, we will recruit up to 24 patients, with up to twelve who speak English and up to twelve who speak Urdu or Punjabi.

Recruitment and Eligibility

Participants will be identified from TB clinics in the English Midlands by members of their normal care team.

Patients will be considered eligible if they satisfy all the following criteria:

- aged 65 years or older at the time of diagnosis
- any gender
- resident in the catchment area
- English, Urdu or Punjabi speakers.
- receiving treatment for active TB
- managed in the community
- able to give informed consent
- hear well enough to have a remote conversation.

The case definition for active TB is:
1. Culture of a sample has resulted in the growth of any of the *M. tuberculosis* complex of organisms, OR.

For peer review only

2. a clinician's judgement that the patient's clinical condition is compatible with tuberculosis, AND a clinician's decision to treat the patient with a full course of anti-TB therapy.[51]

Strain of TB, resistance or relapses will not influence eligibility.

Urdu and Punjabi were selected from amongst other languages as they are the most frequently spoken non-English languages in our catchment area.

A stratified purposive sampling approach will be taken. Participants will first be stratified by language (English or Urdu/Punjabi speaking) and then gender (See Fig 3.) Within each stratum, there will be purposive sampling to select those with differing sites of disease (pulmonary or extra-pulmonary), a range of delay times and a spectrum of ages over 65 years. To avoid bias, outstanding strata for recruitment will be regularly reviewed with the clinical teams.

Figure 3: Showing planned stratification of participants

Participants will be given the option of taking part in the interview with or without the case note review.

Participants will be offered a £20 voucher for taking part in the study.

Data Collection

Data will be collected from the participant and from their primary care record generating a patient story, a primary care story as recorded in the notes and a secondary care story as recorded in letters.

The interview

Participant experience will be captured using a narrative interview. The interview is expected to last up to one and a half hours, with additional travel time for those being interviewed in person. A modification of the schema for an unstructured, in-depth interview suggested by Jovechelovitch and Bauer, 2000, will be applied.[52]

1. Initiation of the interview. An open question will be asked which defines the story of interest, its start point and its end point.
2. Main narration. During this phase there will be minimal interruptions, with simple verbal and non-verbal cues to encourage the participant.
3. Questioning phase. Key events of interest will be identified prior to the interview and the participant's own words will be used to probe further about these events if they have not already been discussed. However, this will not include seeking opinions or judgements.
4. Concluding talk. During this phase, member checking will be carried out by directly asking participants if they are happy with the information shared.

Interviews in English will be carried out by FK and all Urdu and Punjabi interviews will be supervised by FK. FK has experience in conducting focus groups and has experience of collecting narratives in clinical settings. She has undertaken training in narrative methods.

A topic guide has been developed to ensure that key turning points in the story are captured (Supplementary material 1). This will be tested with one English speaking and one Urdu or Punjabi speaking participant and adapted as needed.

For peer review only

Interviews will be transcribed verbatim in the language of the participant and translated into English where needed. Data will be managed by using NVivo.

Primary Care Records

The eligibility time for extraction from records will start from twelve months prior to the date of symptom onset as reported by participants and will end at the date of treatment initiation as reported by the TB clinic. This long look-back period is chosen since there is limited evidence about errors in recall of onset date.[53]

A list of alert symptoms for TB has been developed for another part of this project. This includes general symptoms such as fever, night sweats and weight loss; site-specific symptoms such as productive cough or enlarged lymph nodes for disease in the lungs or lymph nodes, respectively; and tests commonly carried out in primary care which may be abnormal in TB. Any consultation, within the eligible time, in which these signs, symptoms, or TB disease manifestations uncovered during interviews are mentioned will be extracted. Where a consultation deals with more than one problem, and the TB related entry can be clearly differentiated, then only this entry will be extracted. A frequency count of tests will be noted, but only abnormal tests results will be extracted. Letters to and from secondary care within the eligible time frame which are related to the TB journey will also be extracted.

Relevant consultations will be identified by checking the case notes from the beginning of the eligibility period. This approach was selected as using search functions may miss data where there are misspellings or unusual abbreviations. Date of first presentation, date of referral, frequency of consultations, number of team members consulted, number of secondary care clinics attended, and presence of co-morbidities will be recorded and used to describe participants.

Data extraction within GP surgeries will be carried out by FK. FK has experience in the use of several different primary care electronic health care record systems.

Cross cultural research

There is co-creation of the narrative between the researcher and the narrator; the story told is influenced by the audience.[54] In this study, for 12 of the participants, interpreters will be gathering data with minimal interruptions to translate the content. To aid this process, interpreters will be recruited from amongst bilingual members of the university communities who have research exposure or experience before resorting to use of formal translation services. Interpreters will be given training to cover the rationale and methods used in this project. The decision to restrict the range of languages to only Urdu and Punjabi was taken to ensure maximal chances for recruitment and training of appropriately qualified interpreters. We accept that this decision may result in missing information.

Stories gathered in Urdu or Punjabi will be analysed in English. In order to retain the depth of detail and nuance of the data, we will apply an approach suggested by Lopez *et al*.[55] Where the interview was conducted by an interpreter, the audio recording will be translated by an independent person with the aim of conceptual equivalence. The second translator will be asked to highlight where idioms have been used and what these mean.[56] A university-based interpreter will be asked to review the written translations and compare these to the audio recordings. Any discrepancies will be resolved by discussion with the study team.

Data Analysis

Narrative analysis refers to a collection of different methods with the shared characteristic of analysing narratives as a whole unit. This group of methods was chosen since they allow

For peer review only

investigation of events, the experience of living through those events, the socio-cultural contexts of the participants and the way in which the participant has chosen to tell their story. These features are important in achieving the objectives of this study.

The three data sources (interview, primary care record and secondary care letters) for each patient are distinct stories with different narrators and viewpoints. The main analysis will therefore treat each data source separately. Additionally, based on the Critical Realism mindset which sees each data source as a different tool to capture the *actual* patient journey, we will explore the data through triangulation after matching data sources for individual patients.

We will carry out dialogical narrative analysis using a method recommended by Smith, 2016.[42] Although this is presented as a numbered list, the process is iterative, and steps two to five will be repeated several times:

1. "Indwelling" – this is the process of familiarization by reading the transcript several times.

2. Identifying or smoothing the story – at this stage the "core story" or stories are identified. This enables shortening of the narrative to aid further analysis.

   For the interviews, comments from the interviewer will be removed. The text will be re-arranged to provide a chronological arc to the story. Extraneous material (if any) will be marked in a different colour.[57]

   For the textual data sources portions of the consultations or letters which refer to health problems which are clearly different to TB will be removed. Again, data will be arranged chronologically if needed.

3. Identifying narrative themes – rather than line by line coding, the aim here is to identify themes which run through the whole story. At this stage we are most interested in exploring what is said. Both manifest and latent themes may be present.

4. Identifying the structure of stories – the aim here is to understand "how the story is put together".[42] At this stage we are interested in identifying how things have changed over time. For example, there may be stories of progress or stability over time.

5. Opening up the analysis – at this stage Smith recommends asking a series of questions of the stories.
   a. Resource questions. Why is the person telling this story in this way? What templates of narratives are they drawing on to tell this story?
   b. Circulation questions. Who is the intended audience?
   c. Connection questions. Does this story create a "them" and "us"? Is there someone the storyteller does not want to hear this story?
   d. Identity questions. Does the story give the person a sense of identity? Or does it identify who they might want to be in the future?
   e. Function questions. Does the story help or hinder the storyteller? What effect can or could the story have on other people?

Twenty percent of the data will also be analysed by KS to ensure that no core stories or narrative themes are missed.

For peer review only

The results will be drawn together by building a typology of stories. Stories from within each data source and across the whole data set will be clustered together according to shared characteristics in the domains of themes, structure, resources and function.

Triangulation

The approach to triangulation will follow a method suggested by Farmer *et al*, 2006.[58] Data will be triangulated within participants and across the whole data set by looking for convergence, completeness, silence and dissonance across the domains of narrative themes, story structure, resources and function. This will allow us to understand how the perspectives of the story tellers impact on the story told.

Bringing rigour

Trustworthiness and plausibility[59] will be evidenced through member checking at the end of the interview by asking participants if they are happy with all the material they have shared. The context of interviews and an explanation of data gathering techniques will also be provided in the report.

In order to increase criticality,[59] FK will keep a contemporaneous reflexive diary to help understand her role as a researcher rather than GP, and to explore her own biases and preconceptions.

Recruitment and interviews will take place from April to December 2023 and analysis will be completed by March 2024.

Patient and Public involvement

People with living TB, older adults with chronic conditions and their carers were consulted about the project aims, methods and implementation. This directly influenced the decision to interview participants alone, impacted on the locations for interviews and the methods of dissemination. Patient partners commented on the information leaflets and consent forms. Bilingual members of the public helped to recruit translators.

ETHICS AND DISSEMINATION

Ethics

Some of the key ethical issues raised by the study are discussed below.

Equity

TB often affects the most vulnerable in society such as those living in deprivation or experiencing the effects of substance misuse or time in prison.[16] It is important that we include these voices in our research. TB teams have been sensitised to identification of participants based on the eligibility criteria alone. If there are concerns about violence towards staff or difficulty engaging, participants will be offered remote interviews only.

TB affects migrant populations. Our approach to recruitment means that English speaking migrants from a range of backgrounds can be included. However, we have restricted the number of languages included and this means that some voices will not be heard.

We have made the decision to transcribe all interviews in the language of the participants to allow reporting in the source language and to ensure that all participants' original words are available for secondary analysis.

For peer review only

Recruitment

The duration of TB treatment means that clinical teams and patients may develop strong relationships. To reduce the risk of participants feeling obliged to participate, they will be made aware that non-participation will not negatively affect their care and that they are free to withdraw at any time. Although the clinical teams will identify potential participants, consent will be obtained by the research team.

Harms to participants

Some participants may find it distressing to talk about their experiences. Should this occur, the interview will be paused or terminated. FK is an experienced clinician and has training and experience in de-escalating situations. The nature of narrative interviews means that participants are in control of the level of detail they reveal. Participants will be allowed time to debrief and will be signposted to local support services if required.

Interviews will take place remotely or in clinical spaces and during normal working hours to limit potential environmental harms to both participants and researchers, and to allow prompt responses to expressed clinical needs. Input from our PPI partner suggested that evenings and weekends were valuable free time for those in employment and so should not be used for interviews. This approach may result in recruitment bias against those who are in full time employment. We will report the numbers of those who were unable to take part in the study due to this decision.

Confidentiality

Data will be stored in anonymised, encrypted, electronic formats. IT Security Teams have been consulted to ensure that data storage and transfer procedures are appropriate. Aspects of the narrative which might lead to identification of participants will not be reported.

The patient information leaflet clarifies that there may be situations in which disclosures are needed. In line with General Medical Council, UK, guidelines,[60] participants will first be asked for permission to disclose information. If permission is not forthcoming, then identifiable information will only be shared if this is in the interests of the patient or the public. All disclosures will be noted as part of the participants research record.

Stigma

Erving Goffman described three domains types of stigma, that associated with physical or mental illness (for example TB), that associated with identifying as part of a marginalised group (for example ethnic minorities or being an older adult), or that associated with moral character (for example substance misuse) (quoted in [61]). Our study participants are vulnerable to stigmatisation due to a confluence of one or more of these domains. Following guidelines suggested by Gabbidon and Chenneville, 2020, [61] we are reducing inadvertent stigmatisation by engaging in reflexivity around positionality and consulting with patient partners. Narrative methods give control of the content shared to study participants before allowing probing by the interviewer. We have clarified pathways for additional support should the interviews cause distress. We intend to describe the context and will take care to avoid identification through quotes. Participants will be anonymously acknowledged.

Infection control concerns

Face to face interviews will only take place after the TB team confirm that this is safe. The use of personal protective equipment and distancing measures will be determined by national guidelines, University and NHS Trust policies.

For peer review only

Case note reviews

Accessing case notes may make participants feel exposed or vulnerable. To this end, participants will be able to consent to the interview study alone.

Data extraction will be kept proportionate by using a pre-identified list of symptoms and signs or the participant's own words to identify appropriate material for the study.

Case note review may also make participants' GPs feel exposed. A graded approach will be taken to handling potential risks to participant safety. Where there is an ongoing or active risk, for example monitoring blood tests not being carried out, this will be flagged up using an auditable electronic messaging service.

Where there is the suggestion of historic risk to patient safety, for example avoidable treatment delay, the surgery will be made aware of this as a potential significant event. Preferred routes and recipients of each type of communication will be determined on a surgery-by-surgery basis.

This study has been approved by the National Health Service Health Research Authority and the London and Harrow Ethics Committee in April 2022 (ref: 22/PR/0357).

Dissemination

This study forms part of a larger body work which will be submitted and published as a PhD thesis. Following analysis, a plain language summary will be shared with participants. Findings will be shared in peer reviewed journals and through public facing materials developed in conjunction with TB services, primary care colleagues, patient partners and TB charities.

FUNDING STATEMENT

FK is funded by the NIHR Doctoral Research Fellowship, grant number 300688. The views expressed are those of the author(s) and not necessarily those of the NIHR or the Department of Health and Social Care.

The project is being delivered with support from the Clinical Research Networks for Primary and Secondary Care in the West Midlands.

COMPETING INTERESTS STATEMENT

There are no competing interests to declare.

AUTHOR CONTRIBUTIONS

FK conceived the study with support from KS and NMc

FK wrote the first draft of the paper and all authors commented on revisions.

DATA STATEMENT SECTION

Interview transcripts will be stored for ten years after the end of the study and will made available for secondary analysis on a case-by-case basis.

For peer review only

REFERENCES

1. Agency, U.H.S. *Tuberculosis in England 2022 report (data up to the end of 2021)*. 2022 June 2023 [cited 2023; Available from: https://www.gov.uk/government/publications/tuberculosis-in-england-2022-report-data-up-to-end-of-2021/tb-incidence-and-epidemiology-in-england-2021.
2. Agency, U.H.S., *TB Action Plan for England, 2021 to 2026*. 2021: London.
3. Greenaway, C., et al., *Delay in Diagnosis among Hospitalized Patients with Active Tuberculosis—Predictors and Outcomes.* American Journal of Respiratory and Critical Care Medicine, 2002. **165**(7): p. 927-933.
4. Virenfeldt, J., et al., *Treatment delay affects clinical severity of tuberculosis: a longitudinal cohort study.* BMJ Open, 2014. **4**.
5. Cheng, S., et al., *Effect of Diagnostic and Treatment Delay on the Risk of Tuberculosis Transmission in Shenzhen, China: An Observational Cohort Study, 1993–2010.* PLOS ONE, 2013. **8**(6): p. e67516.
6. Golub, J.E., et al., *Delayed tuberculosis diagnosis and tuberculosis transmission.* The International Journal of Tuberculosis and Lung Disease, 2006. **10**(1): p. 24-30.
7. Melsew, Y.A., et al., *Risk factors for infectiousness of patients with tuberculosis: a systematic review and meta-analysis.* Epidemiology and Infection, 2018. **146**(3): p. 345-353.
8. NICE. *NICE Guideline 33 - Tuberculosis*. 2016 27.08.2018]; Available from: https://www.nice.org.uk/guidance/ng33.
9. Nnoaham, K.E., et al., *Perceptions and experiences of tuberculosis among African patients attending a tuberculosis clinic in London.* The International Journal of Tuberculosis and Lung Disease, 2006. **10**(9): p. 1013-1017.
10. Public Health England, *Tuberculosis in England 2015 Report: presenting data to the end of 2014* 2015.
11. Public Health England, *Tuberculosis in England 2016 report: presenting data to end of 2015.* 2016, PHE.
12. Public Health England, *Tuberculosis in England 2017 report (presenting data to end of 2016).* 2017.
13. Public Health England, *Tuberculosis in England 2018 report. Presenting data to end of 2017.* 2018: PHE publications.
14. Public health England, *Tuberculosis in England 2019 report (presenting data to end of 2018).* 2019: PHE, London.
15. Public Health England, *Tuberculosis in England 2020 report presenting data to end of 2019.* 2020, PHE.
16. UK Health Security Agency, *Tuberculosis in England. 2021 report (Presenting data to end 2020)*. 2021, UKHSA: UK.
17. Broderick, C., et al., *Delays in the diagnosis and treatment of bone and joint tuberculosis in the United Kingdom.* The Bone & Joint Journal, 2018. **100-B**(1): p. 119-124.
18. Abbara, A., et al., *Time to diagnosis of tuberculosis is greater in older patients: a retrospective cohort review.* ERJ open research, 2019. **5**(4): p. 00228-2018.
19. Walter, F., et al., *The Andersen Model of Total Patient Delay: a systematic review of its application in cancer diagnosis.* Journal of health services research & policy, 2012. **17**(2): p. 110-118.
20. Wieland, M.L., et al., *Perceptions of tuberculosis among immigrants and refugees at an adult education center: a community-based participatory research approach.* Journal of immigrant and minority health, 2012. **14**(1): p. 14-22.
21. Gerrish, K., A. Naisby, and M. Ismail, *The meaning and consequences of tuberculosis among Somali people in the United Kingdom.* Journal of Advanced Nursing, 2012. **68**(12): p. 2654-2663.

22. Craig, G.M., A. Joly Lm Fau - Zumla, and A. Zumla, *'Complex' but coping: experience of symptoms of tuberculosis and health care seeking behaviours--a qualitative interview study of urban risk groups, London, UK.* BMC Public Health, (1471-2458 (Electronic)).
23. Quattrocchi, A., et al., *Determinants of patient and health system delay among Italian and foreign-born patients with pulmonary tuberculosis: a multicentre cross-sectional study.* BMJ Open, 2018. **8**.
24. Asch, S., et al., *Why Do Symptomatic Patients Delay Obtaining Care for Tuberculosis?* American Journal of Respiratory and Critical Care Medicine, 1998. **157**(4): p. 1244-1248.
25. Calder, L., W. Gao, and G. Simmons, *Tuberculosis: reasons for diagnostic delay in Auckland.* N Z Med J, 2000. **113**: p. 483.
26. Peri, A.M., et al., *Determinants of patient and health care services delays for tuberculosis diagnosis in Italy: a cross-sectional observational study.* BMC Infectious Diseases, 2018. **18**(1): p. 690.
27. Abarca Tomás, B., et al., *Tuberculosis in Migrant Populations. A Systematic Review of the Qualitative Literature.* PLOS ONE, 2013. **8**(12): p. e82440.
28. Roberts, D.J., et al., *Factors associated with delay in treatment initiation for pulmonary tuberculosis.* ERJ Open Research, 2020. **6**.
29. Wyman, M.F., S. Shiovitz-Ezra, and J. Bengel, *Ageism in the Health Care System: Providers, Patients, and Systems*, in *Contemporary Perspectives on Ageism*, L. Ayalon and C. Tesch-Römer, Editors. 2018, Springer International Publishing: Cham. p. 193-212.
30. Jones, D., et al., *Factors influencing symptom appraisal and help-seeking of older adults with possible cancer: a mixed-methods systematic review.* British Journal of General Practice, 2022. **72**(723): p. e702.
31. Teo, K., et al., *Help-Seeking Behaviors Among Older Adults: A Scoping Review.* Journal of Applied Gerontology, 2022. **41**(5): p. 1500-1510.
32. Paynter, S., et al., *Patient and health service delays in initiating treatment for patients with pulmonary tuberculosis: retrospective cohort study.* The International Journal of Tuberculosis and Lung Disease, 2004. **8**(2): p. 180-185.
33. Loutet, M.G., et al., *Delay from symptom onset to treatment start among tuberculosis patients in England, 2012–2015.* Epidemiology and Infection, 2018. **146**(12): p. 1511-1518.
34. Tattevin, P., et al., *Factors associated with patient and health care system delay in the diagnosis of tuberculosis in France.* The International Journal of Tuberculosis and Lung Disease, 2012. **16**(4): p. 510-515.
35. Saldana, L., et al., *Factors affecting delay in initiation of treatment of tuberculosis in the Thames Valley, UK.* (1476-5616 (Electronic)).
36. Farah, M.G., et al., *Patient and health care system delays in the start of tuberculosis treatment in Norway.* BMC infectious diseases, 2006. **6**: p. 33-33.
37. Sultan, H., S. Haroon, and N. Syed, *Delay and completion of tuberculosis treatment: a cross-sectional study in the West Midlands, UK.* Journal of Public Health, 2013. **35**(1): p. 12-20.
38. NIHR. *NIHR Active awards*. 2020 [cited 2023; Available from: https://dev.fundingawards.nihr.ac.uk/award/NIHR300688.
39. Alderson, P., *Critical realism for health and illness research: A practical introduction*. 2021: Policy Press.
40. Koopmans, E. and D.C. Schiller, *Understanding Causation in Healthcare: An Introduction to Critical Realism.* Qualitative Health Research, 2022. **32**(8-9): p. 1207-1214.
41. Greenhalgh, T. and B. Hurwitz, *Why study narrative?* BMJ, 1999. **318**: p. 48-50.
42. Smith B, *Narrative Analysis*, in *Analysing Qualitative Data in Psychology*, Lyons E and C. A, Editors. 2016, Sage: London.
43. Connelly, F.M. and D.J. Clandinin, *Stories of Experience and Narrative Inquiry.* Educational Researcher, 1990. **19**(5): p. 2-14.

For peer review only

44. Burck, C., *Comparing qualitative research methodologies for systemic research: the use of grounded theory, discourse analysis and narrative analysis.* Journal of Family Therapy, 2005. **27**(3): p. 237-262.
45. Hsu, M.Y. and B. McCormack, *Using narrative inquiry with older people to inform practice and service developments.* Journal of Clinical Nursing, 2012. **21**(5-6): p. 841-849.
46. Schwind, J.K., et al., *What can be learned from patient stories about living with the chronicity of heart illness? A narrative inquiry.* Contemporary Nurse, 2016. **52**(2-3): p. 216-229.
47. Sim, J., et al., *Can sample size in qualitative research be determined a priori?* International Journal of Social Research Methodology, 2018. **21**(5): p. 619-634.
48. Vasileiou, K., et al., *Characterising and justifying sample size sufficiency in interview-based studies: systematic analysis of qualitative health research over a 15-year period.* BMC Medical Research Methodology, 2018. **18**(1): p. 148.
49. Guest, G., A. Bunce, and L. Johnson, *How Many Interviews Are Enough?:An Experiment with Data Saturation and Variability.* Field Methods, 2006. **18**(1): p. 59-82.
50. Hennink, M.M., B.N. Kaiser, and V.C. Marconi, *Code Saturation Versus Meaning Saturation:How Many Interviews Are Enough?* Qualitative Health Research, 2017. **27**(4): p. 591-608.
51. Public Health England. *Tuberculosis: notifying cases*. 2014 2015 Jan 2021]; Available from: https://www.gov.uk/government/publications/tuberculosis-notifying-cases.
52. Jovchelovitch, S. and M.W. Bauer. *Narrative interviewing {online}*. originally published in Bauer, Martin W.; Gaskell, G.; Eds., Qualitative researching with text, image and sound : a practical handbook. London, England: SAGE Publications, 2000. 2000 15.05.2020]; Available from: http://eprints.lse.ac.uk/2633
53. Schmier, J.K. and M.T. Halpern, *Patient recall and recall bias of health state and health status.* Expert Review of Pharmacoeconomics & Outcomes Research, 2004. **4**(2): p. 159-163.
54. Haydon, G. and P. van der Riet, *Narrative inquiry: A relational research methodology suitable to explore narratives of health and illness.* Nordic Journal of Nursing Research, 2016. **37**(2): p. 85-89.
55. Lopez, G.I., et al., *Translation Barriers in Conducting Qualitative Research With Spanish Speakers.* Qualitative Health Research, 2008. **18**(12): p. 1729-1737.
56. Wong, J.P.-H. and M.K.-L. Poon, *Bringing Translation Out of the Shadows: Translation as an Issue of Methodological Significance in Cross-Cultural Qualitative Research.* Journal of Transcultural Nursing, 2010. **21**(2): p. 151-158.
57. Emden, C., *Conducting a narrative analysis.* Collegian, 1998(1322-7696 (Print)).
58. Farmer, T., et al., *Developing and Implementing a Triangulation Protocol for Qualitative Health Research.* Qualitative Health Research, 2006. **16**(3): p. 377-394.
59. Greenhalgh, T., *Cultural contexts of health: the use of narrative research in the health sector*, in *Health Evidence Network (HEN) synthesis report*. 2016, WHO Regional Office for Europe: Copenhagen.
60. Council, G.M. *Ethical and legal duties of confidentiality*. 2020 30.07.2020]; Available from: https://www.gmc-uk.org/ethical-guidance/ethical-guidance-for-doctors/confidentiality/disclosures-for-the-protection-of-patients-and-others.
61. Gabbidon, K. and T. Chenneville, *Strategies to minimize further stigmatization of communities experiencing stigma: A guide for qualitative researchers.* Stigma and Health, 2021. **6**(1): p. 32-42.

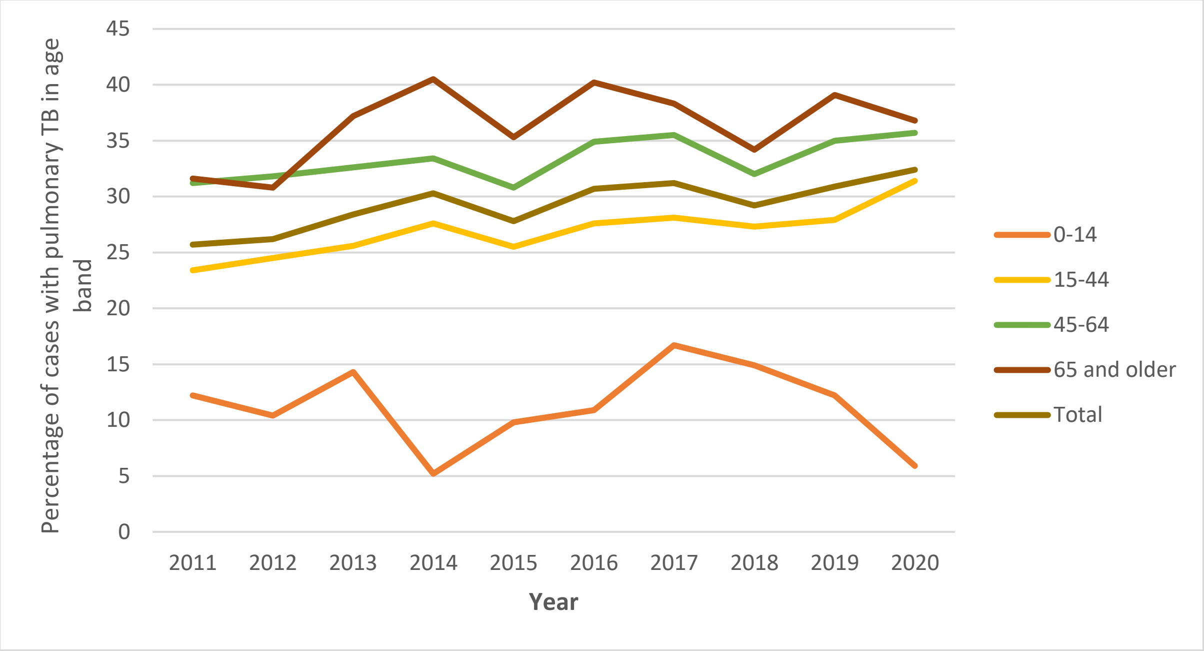

For peer review only

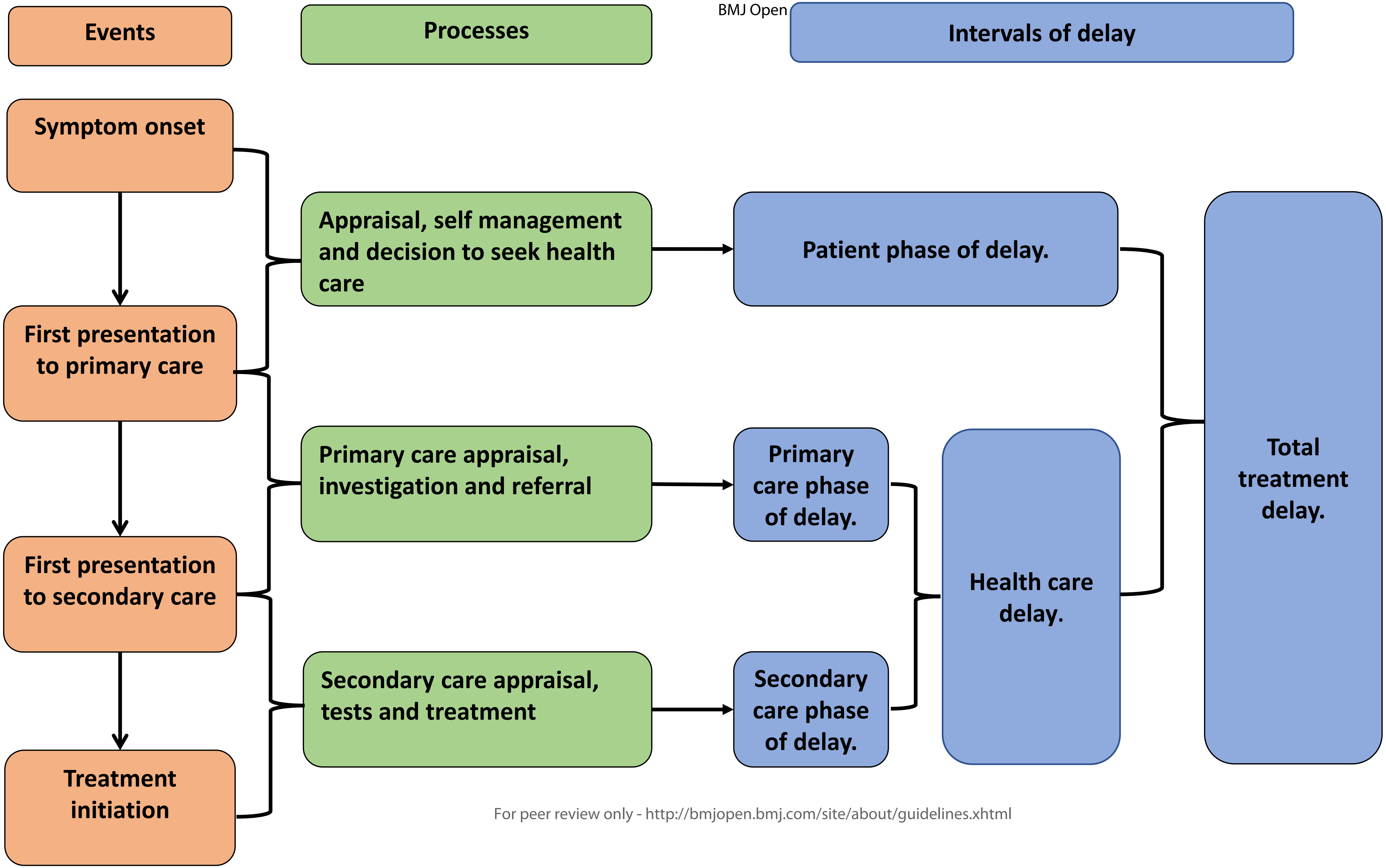
Events
Processes
Intervals of delay
Symptom onset
Appraisal, self management and decision to seek health care
Patient phase of delay.
First presentation to primary care
Primary care appraisal, investigation and referral
Primary care phase of delay.
Health care delay.
Total treatment delay.
First presentation to secondary care
Secondary care appraisal, tests and treatment
Secondary care phase of delay.
Treatment initiation

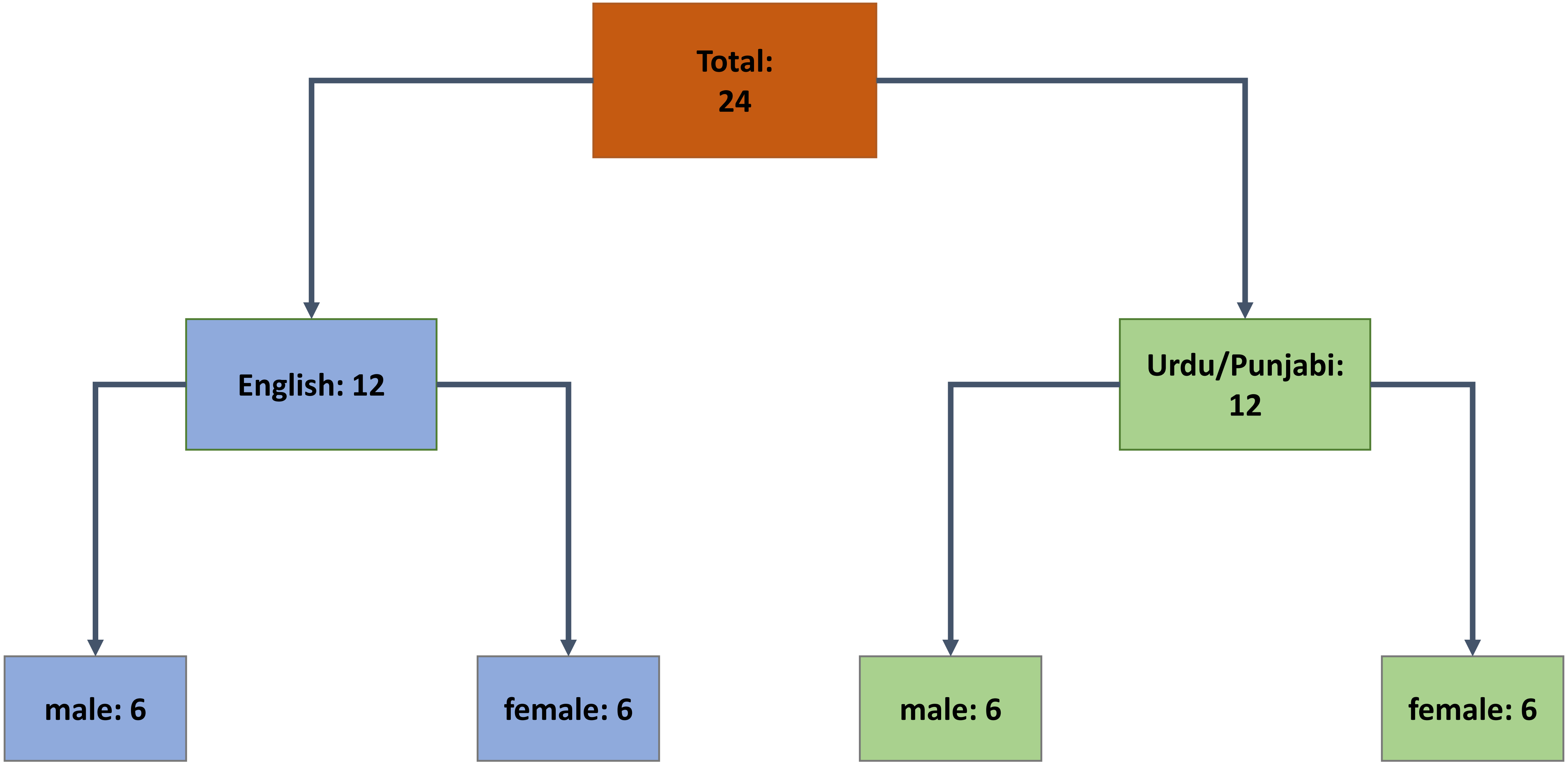
Total:
24
English: 12
Urdu/Punjabi:
12
male: 6
female: 6
male: 6
female: 6

Supplementary Material

From symptom onset to treatment initiation: A narrative study of Older Adults with Tuberculosis (OAT)

The OAT Study

Topic Guide

(Date 20/09/2020 Version 1.0)

For peer review only

The OAT Study will take a narrative approach to data gathering. The guide will the trialled on English and non-English speaking participants and adapted if needed.

Step One: Introductions, consent and identity confirmation

- Introduce self and confirm that the call is to take consent and carry out the interview.
- Ask participant to confirm their name and date of birth.
- Confirm that this is a suitable time.
- Ask participant if they have any questions about the information sheets, consent form or study in general.
- Thanks for agreeing to participate.
- Advise re likely duration: about one hour
- Check if there is anyone else in the room. Confirm that the participant is happy for that person to be in room. Ask that others in the room do not interject during the interview and advise them that it may not be possible to remove their comments from the recordings, but that these comments will not be used for the research. Be prepared to comment during the interview if there are too many interruptions.
- Explain the format of the interview – ie
    - participant invited to tell their story.
    - Define the scope – from time of onset of illness to treatment initiation.
    - "Once you have finished telling your story, I might ask you some questions."
- Advise the participant that recording will begin and turn recording devices on.
- State
    - "This is the audio recorded consent for the OAT Study".
    - For "PARTICPANT NAME" and "DATE OF BIRTH".
    - Recorded on "DATE" by "INVESTIGATOR'S NAME".
    - "I am going to read each statement from Consent Form Two. If you agree with the statement, please say "I agree". You must agree with all the statements to be eligible to take part in the study. Your consent will be audio recorded and stored securely."
    - Read each statement from Consent Form Two. Ensure that participant's agreement to each statement is clearly heard.

For peer review only - http://bmjopen.bmj.com/site/about/guidelines.xhtml

Step Two: Initiation of the interview and main narration

- Advise participant that interview will begin
- "I am interested in trying to understand your experience of being diagnosed with TB. Can you tell me what happened and how you felt from the time you first became unwell until you started treatment for TB?"
- Take notes and use verbal and non-verbal prompts.
- When the narrative reaches a natural end, ask, "Is there something else you want to tell me?"

Step Three: Questioning Phase.

Questions should be formulated using the participant's own words. The questions below are a guide only.

Questions should not start with "Why" and should not be used to point out contradictions in the narrative.

Points which should be probed if they have not been covered in the narrative. Every point will not be relevant to all participants:

- Generally applicable question structure, "You mentioned XXX, can you say a bit more about that?"
- Is symptom onset clearly identified. For example, "How were you feeling before "FIRST SYMPTOMS" started?"
- Appraisal process. For example, "What made you realise that you were not well?"
- Symptoms before presenting to health care. For example, "How were you feeling before you went to see the doctor or nurse?"
- Trigger to seek health care. For example, "What made you decide to go and see the doctor or nurse?"
- Influence of others on the decision to seek health care. For example, "Did friends or family encourage or discourage you from seeing a doctor or nurse?"
- Ease of access to health care. For example, "Was it easy to get an appointment to see a doctor or nurse?"
- Experience of first and subsequent contacts with health care. For example, "What happened when you first saw a doctor or nurse?" and/ or, "How did you feel after you went to see the doctor or nurse?"
- Experience of waiting for a diagnosis. For example, "How did you feel while you were waiting for a diagnosis?"
- Experience of getting a diagnosis. For example, "What did it feel like when you found out you have TB?"
- Experience of receiving treatment. "Can you tell me more about the experience of receiving treatment?"
- Impact of COVID. "How did COVID affect you and your care?"

Step Four: Concluding talk

- Ask participant if they are happy with the information shared
- Advise the participant that the recording is ending.

For peer review only - http://bmjopen.bmj.com/site/about/guidelines.xhtml

- Note additional information received after the recording is completed as contextual information in field notes. This will not be included in the analysis.

For peer review only

For peer review only - http://bmjopen.bmj.com/site/about/guidelines.xhtml

BMJ Open

From symptom onset to treatment initiation: protocol for a narrative study exploring the journey of older adults with tuberculosis in the English Midlands, UK

Journal:	*BMJ Open*
Manuscript ID	bmjopen-2022-070933.R2
Article Type:	Protocol
Date Submitted by the Author:	15-Sep-2023
Complete List of Authors:	Kidy, Farah; University of Warwick Warwick Medical School<br>McCarthy, Noel; Trinity College Dublin<br>Seers, Kate; University of Warwick Warwick Medical School, RCN RI
**Primary Subject Heading**:	Public health
Secondary Subject Heading:	Epidemiology, General practice / Family practice, Infectious diseases, Qualitative research
Keywords:	Tuberculosis < INFECTIOUS DISEASES, Quality in health care < HEALTH SERVICES ADMINISTRATION & MANAGEMENT, Public health < INFECTIOUS DISEASES, PRIMARY CARE

SCHOLARONE™
Manuscripts

From symptom onset to treatment initiation: protocol for a narrative study exploring the journey of older adults with tuberculosis in the English Midlands, UK

Farah Kidy[1]

Noel McCarthy[2]

Kate Seers[1]

Corresponding author:

Farah Kidy, f.kidy@warwick.ac.uk

[1] Warwick Medical School, University of Warwick, Gibbet Hill Road, Coventry, UK

[2] School of Medicine, Trinity College Dublin, College Green, Dublin 2, Ireland

For peer review only

Word count, excluding title page, abstract, references, figures and tables: 3996

KEY WORDS: Tuberculosis, treatment delay, older adults, narrative inquiry, case note review

ABSTRACT

Introduction

Time from symptom onset to treatment initiation in tuberculosis (TB) remains stubbornly prolonged despite reductions in disease incidence. Delays may contribute to increased morbidity, mortality, onward spread of disease and poor patient experiences. Most delays occur prior to hospital referral. The average primary care healthcare provider in England is unlikely to see TB on a regular basis. Little is known about primary care diagnostic and referral challenges.

Adults aged 65 years or older are more likely to experience delays. However, little is known about their journey from symptom onset to treatment initiation.

Methods and analysis

We will carry out a narrative study including adults aged 65 years or older, living in the English Midlands and receiving treatment for active TB. Twelve English and 12 Urdu or Punjabi speakers will be recruited from TB clinics and interviewed. Their primary care records will be accessed, and the primary care story and secondary care letters will be extracted. Each of the data sources will be analysed using dialogical narrative analysis. Data will be triangulated within participants and across the data set.

Ethics and dissemination

This study received approval from the Health Research Authority and the Research Ethics Committee in April 2022. Risk management and equity considerations have been made a priority. Findings will be disseminated through publication in open access peer reviewed journals, presentations to policy makers, primary healthcare and secondary healthcare professionals, and through public facing materials developed in conjunction with patients, members of the pubic, TB services and charities.

ARTICLE SUMMARY

Strengths and limitations of this study

- Using narrative methods allows for an understanding of the sequence and experience of events and opens the door to exploring causal mechanisms.
- This study will recruit participants whose experiences are seldom considered and who are most likely to suffer from negative consequences of tuberculosis.
- Including only Urdu, Punjabi and English speakers is a potential limitation.
- Case notes may be challenging to analyse and interpret.

INTRODUCTION

England is a low-incidence tuberculosis (TB) country with an incidence of 7.8 per 100,000 in 2021.[1] Despite decreases in the incidence of TB since 2011, time from symptom onset to treatment initiation (treatment delay) remains stubbornly prolonged. Neither the proportion of people waiting (31.6% waited for 4 months or longer in 2021), nor the median delay (79 days, inter-quartile range 40-153) have improved in the last five years.[1]

The TB Action Plan for England sets out the priorities and actions for TB services, the UK Health Security Agency (UKHSA), the NHS in England and other stakeholders to ensure that

For peer review only

the country meets its commitment to eliminate TB as a public health problem by 2035.[2] Recovery from Covid and prevention, detection and control of TB are prioritised. Addressing treatment delays underpins activities within all these areas.

Treatment delays are a particular focus for action because they may lead to harmful consequences including increased morbidity,[3-5] mortality,[3] transmission,[3, 6, 7] and poor patient experiences [8, 9].

Adults aged 65 years or older contribute 16% of cases with pulmonary TB but make greater contribution to those who experience delays of 4 months or longer.[10-16] Older adults are also more likely to develop extra-pulmonary disease which is associated with even longer delays (median 9 months in orthopaedic disease).[8, 17, 18] A greater proportion of this age group require additional support during treatment (37% vs 32.9% of 45 to 64 year olds, or 28.2% of 15 to 44 year olds).[1] This support ranges from additional contact with TB nurses to directly observed therapy. They are also less likely to complete treatment than their younger counterparts (65.6% vs 84.8%, or 88.3% respectively).[1]

Research to reduce treatment delays is most advanced in cancer studies. Here a decade of experience highlights the importance of exploring the whole patient journey. From the process of self-appraisal which identifies that there has been a shift from the norm, to the decision to seek healthcare and subsequent actions of healthcare professionals.[19] A modified version of Walter *et al*'s, 2012, model provides a framework to reflect on as the study progresses, Fig 1.

Figure 1: Showing events, processes and intervals of delay in the patient journey from symptom onset to treatment initiation.

The patient phase of delay

There is a body work (using methods such as focus groups[20], interviews[9, 21, 22], and surveys or questionnaires[23-26]) exploring healthcare seeking behaviours in adults with TB in low incidence countries. Perceived stigma and fear of being isolated from loved ones were barriers,[9, 20, 23] particularly amongst migrant populations.[9, 21, 23, 27] In some cases, mild symptoms were misinterpreted as being due to something which would resolve itself or could be treated at home.[9, 22, 24-26] In some settings, practical considerations such as distance to healthcare facility, having to pay for transport or anticipated wait times were barriers.[23, 24, 27] Being a new migrant or facing language barriers delayed presentation to healthcare services.[26, 28] Unfortunately, few participants aged 65 or older were included in these studies.

There is a long-standing argument that older adults experience ageism at individual, policy and system levels when engaging with healthcare.[29] The evidence about self-appraisal and healthcare seeking decisions in older adults is mixed. In a review of cancer studies, age was found to be associated with both increased and decreased patient phase delays.[30] The appraisal process was influenced by symptom awareness and interpretation.[30, 31] The decision to seek healthcare was influenced by the support of others, independence,

For peer review only

awareness of systems, fear of potential diagnoses and self-management including alternative medicine.[30, 31] These influences could both prompt or delay seeking healthcare. These reviews did not include participants with TB.

The healthcare phase of delay

Studies from Europe and the UK have shown greater relative contribution of either patient[32, 33] or healthcare[26, 34-37] stages of treatment delay. In our context, TB is diagnosed in secondary care settings (i.e. outpatient specialist clinics or during an inpatient admission).Most studies did not differentiate between the stages of healthcare delay, but Roberts et al, 2020, report that most healthcare delay occurred prior to referral to secondary care.[28]

The knowledge gaps

To influence patient journeys, we need to understand both what happened and why it happened. This knowledge is missing for older adults with TB from patient, primary and secondary care perspectives. The objective of this study is to address these knowledge gaps by exploring:

1. The sequence of events leading from a point of no new illness to the point of treatment initiation.
2. The experience of living through these events.
3. The impact of others (e.g., family and healthcare providers) on the sequence and experience of these events
4. Recognition in and referral from primary care
5. Recognition and diagnosis in secondary care

METHODS AND ANALYSIS

Ontology and epistemology

Bhaskar, the founder of Critical Realism, offers a three-level description of reality. The *empirical* level is that which is observable, the *actual* level is the reality which exists irrespective of our observations and about which we may or may not be aware and lastly, the *real* level which encompasses unseen causal influences.[38] We assume that there is an *actual* patient journey from symptom onset to treatment initiation, but that our description and understanding of this will vary depending on the method of inquiry used and to whom this is delivered. Explicit acknowledgement of the *real* level allows us to delve into questions about causal mechanisms and how or when they are triggered within complex systems.[39]

Methodology

This study will use narrative inquiry applied to interviews and written records. Data are collected in the form of narratives (or stories) and the whole narrative is the unit of analysis. A narrative has a beginning followed by a sequence of unfolding events, culminating with an end.[40] There is a plot and there are characters other than the narrator.[41] Narratives tell us how humans experience and make sense of the world.[42] Listening to the whole story allows us insight not only into what has happened but also why it happened and what that felt like. There is an explicit focus on understanding how the illness fits into patients' lives.[43] Narrative approaches have been used successfully to explore healthcare

For peer review only

experiences of older adults[44] including those with a different first language to the investigators.[45]

Narrative methods will work well for our population since they allow participants to tell the story that they think is important when they are neither seeking healthcare nor are there any negative consequences to their stories.

Narrative inquiry fits well within Critical Realism as there is an assumption of an *actual* patient journey with people, structures and events. We are *empirically* describing this from both participants' memories and their health records. We will use the content and structure of the narratives to come to conclusions about the *real* mechanisms underlying healthcare seeking behaviour and arrival at diagnoses.

Sample size

Some researchers believe that *a priori* determination of sample sizes is not possible[46] rather that data saturation or data adequacy be used.[47] Others suggest that for non-probabilistic sampling in homogenous populations, most themes are apparent after six interviews and that saturation is reached after 12 interviews.[48] However, Hennick et al, 2017, distinguish between code saturation (heard it all) and meaning saturation (understood it all).[49] With the aim of ensuring meaning saturation of the main narrative themes, we will recruit up to 24 patients, with up to twelve who speak English and up to twelve who speak Urdu or Punjabi.

Recruitment and Eligibility

Participants will be identified from TB clinics in the English Midlands by members of their normal care team.

Patients will be considered eligible if they satisfy all the following criteria:

- aged 65 years or older at the time of diagnosis
- any gender
- resident in the catchment area
- English, Urdu or Punjabi speakers.
- receiving treatment for active TB
- managed in the community
- able to give informed consent
- hear well enough to have a remote conversation.

The case definition for active TB is:
1. Culture of a sample has resulted in the growth of any of the *M. tuberculosis* complex of organisms, OR.

2. a clinician's judgement that the patient's clinical condition is compatible with tuberculosis, AND a clinician's decision to treat the patient with a full course of anti-TB therapy.[50]

Strain of TB, resistance or relapses will not influence eligibility.

Urdu and Punjabi were selected from amongst other languages as they are the most frequently spoken non-English languages in our catchment area.

A stratified purposive sampling approach will be taken. Participants will first be stratified by language (English or Urdu/Punjabi speaking) and then gender (See Fig 2.) Within each

For peer review only

stratum, there will be purposive sampling to select those with differing sites of disease (pulmonary or extra-pulmonary), a range of delay times and a spectrum of ages over 65 years. To avoid bias, outstanding strata for recruitment will be regularly reviewed with the clinical teams.

Figure 2: Showing planned stratification of participants

Participants will be given the option of taking part in the interview with or without the case note review.

Participants will be offered a £20 voucher for taking part in the study.

Recruitment will take place from April to December 2023.

Data Collection

Data will be collected from the participant and from their primary care record generating a patient story, a primary care story as recorded in the notes and a secondary care story as recorded in letters.

The interview

Participant experience will be captured using a narrative interview. The interview is expected to last up to one and a half hours, with additional travel time for those being interviewed in person. A modification of the schema for an unstructured, in-depth interview suggested by Jovechelovitch and Bauer, 2000, will be applied.[51]

1. Initiation of the interview. An open question will be asked which defines the story of interest, its start point and its end point.
2. Main narration. During this phase there will be minimal interruptions, with simple verbal and non-verbal cues to encourage the participant.
3. Questioning phase. Key events of interest will be identified prior to the interview and the participant's own words will be used to probe further about these events if they have not already been discussed. However, this will not include seeking opinions or judgements.
4. Concluding talk. During this phase, member checking will be carried out by directly asking participants if they are happy with the information shared.

Interviews in English will be carried out by FK. Interviews conducted in Urdu and Punjabi will be carried out by trained, research aware members of university staff and trained post-graduate students. If these individuals are not available, named individuals from formal translation services will be trained and engaged for interviews. All interviews will be supervised by FK. FK has experience in conducting focus groups and has experience of collecting narratives in clinical settings. She has undertaken training in narrative methods.

A topic guide has been developed to ensure that demographic details and personal characteristics (such as employment status, living situation and country of birth) as well as key turning points in the story are captured (Supplementary material 1). This will be tested with one English speaking and one Urdu or Punjabi speaking participant and adapted as needed.

Interviews will be transcribed verbatim in the language of the participant and translated into English where needed. Data will be managed by using NVivo.

Primary Care Records

For peer review only

The eligibility time for extraction from records will start from twelve months prior to the date of symptom onset as reported by participants and will end at the date of treatment initiation as reported by the TB clinic. This long look-back period is chosen since there is limited evidence about errors in recall of onset date.[52]

A list of alert symptoms for TB has been developed for another part of this project. This includes general symptoms such as fever, night sweats and weight loss; site-specific symptoms such as productive cough or enlarged lymph nodes for disease in the lungs or lymph nodes, respectively; and tests commonly carried out in primary care which may be abnormal in TB. Any consultation, within the eligible time, in which these signs, symptoms, or TB disease manifestations uncovered during interviews are mentioned will be extracted. Where a consultation deals with more than one problem, and the TB related entry can be clearly differentiated, then only this entry will be extracted. A frequency count of tests will be noted, but only abnormal tests results will be extracted. Letters to and from secondary care within the eligible time frame which are related to the TB journey will also be extracted.

Relevant consultations will be identified by checking the case notes from the beginning of the eligibility period. This approach was selected as using search functions may miss data where there are misspellings or unusual abbreviations. Date of first presentation, date of referral, frequency of consultations, number of team members consulted, number of secondary care clinics attended, and presence of co-morbidities will be recorded and used to describe participants.

Data extraction within GP surgeries will be carried out by FK. FK has experience in the use of several different primary care electronic healthcare record systems.

Data collection (interviews and case note reviews) will take place from April to December 2023.

Cross cultural research

There is co-creation of the narrative between the researcher and the narrator; the story told is influenced by the audience.[53] In this study, for 12 of the participants, interpreters will be gathering data with minimal interruptions to translate the content. To aid this process, interpreters have been recruited from amongst bilingual, research aware members of the university community. The decision to restrict the range of languages to only Urdu and Punjabi was taken to ensure maximal chances for recruitment and training of appropriately qualified interpreters. We accept that this decision may result in missing information, particularly in ethnic minority groups.

Stories gathered in Urdu or Punjabi will be analysed in English. In order to retain the depth of detail and nuance of the data, we will apply an approach suggested by Lopez *et al*.[54] Where the interview was conducted by an interpreter, the audio recording will be translated by an independent person with the aim of conceptual equivalence. The second translator will be asked to highlight where idioms have been used and what these mean.[55] A university-based interpreter will be asked to review the written translations and compare these to the audio recordings. Any discrepancies will be resolved by discussion with the study team.

Data Analysis

Narrative analysis refers to a collection of different methods with the shared characteristic of analysing narratives as a whole unit. This group of methods was chosen since they allow investigation of events, the experience of living through those events, the socio-cultural

For peer review only

contexts of the participants and the way in which the participant has chosen to tell their story. These features are important in achieving the objectives of this study.

The three data sources (interview, primary care record and secondary care letters) for each patient are distinct stories with different narrators and viewpoints. The main analysis will therefore treat each data source separately. Additionally, based on the Critical Realism mindset which sees each data source as a different tool to capture the *actual* patient journey, we will explore the data through triangulation after matching data sources for individual patients.

We will carry out dialogical narrative analysis using a method recommended by Smith, 2016.[41] Although this is presented as a numbered list, the process is iterative, and steps two to five will be repeated several times:

1. "Indwelling" – this is the process of familiarization by reading the transcript several times.

2. Identifying or smoothing the story – at this stage the "core story" or stories are identified. This enables shortening of the narrative to aid further analysis.

   For the interviews, comments from the interviewer will be removed. The text will be re-arranged to provide a chronological arc to the story. Extraneous material (if any) will be marked in a different colour.[56]

   For the textual data sources portions of the consultations or letters which refer to health problems which are clearly different to TB will be removed. Again, data will be arranged chronologically if needed.

3. Identifying narrative themes – rather than line by line coding, the aim here is to identify themes which run through the whole story. At this stage we are most interested in exploring what is said. Both manifest and latent themes may be present.

4. Identifying the structure of stories – the aim here is to understand "how the story is put together".[41] At this stage we are interested in identifying how things have changed over time. For example, there may be stories of progress or stability over time.

5. Opening up the analysis – at this stage Smith recommends asking a series of questions of the stories.
   a. Resource questions. Why is the person telling this story in this way? What templates of narratives are they drawing on to tell this story?
   b. Circulation questions. Who is the intended audience?
   c. Connection questions. Does this story create a "them" and "us"? Is there someone the storyteller does not want to hear this story?
   d. Identity questions. Does the story give the person a sense of identity? Or does it identify who they might want to be in the future?
   e. Function questions. Does the story help or hinder the storyteller? What effect can or could the story have on other people?

Twenty percent of the data will also be analysed by KS to ensure that no core stories or narrative themes are missed.

For peer review only

The results will be drawn together by building a typology of stories. Stories from within each data source and across the whole data set will be clustered together according to shared characteristics in the domains of themes, structure, resources and function.

Triangulation

The approach to triangulation will follow a method suggested by Farmer *et al*, 2006.[57] Data will be triangulated within participants and across the whole data set by looking for convergence, completeness, silence and dissonance across the domains of narrative themes, story structure, resources and function. This will allow us to understand how the perspectives of the story tellers impact on the story told.

Analysis will be completed by March 2024.

Bringing rigour

Trustworthiness and plausibility[58] will be evidenced through member checking at the end of the interview by asking participants if they are happy with all the material they have shared. The context of interviews and an explanation of data gathering techniques will also be provided in the report.

In order to increase criticality,[58] FK will keep a contemporaneous reflexive diary to help understand her role as a researcher rather than GP, and to explore her own biases and preconceptions.

Patient and Public involvement

People with living TB, older adults with chronic conditions and their carers were consulted about the project aims, methods and implementation. This directly influenced the decision to interview participants alone, impacted on the locations for interviews and the methods of dissemination. Patient partners commented on the information leaflets and consent forms. Bilingual members of the public helped to recruit translators.

ETHICS AND DISSEMINATION

Ethics

Some of the key ethical issues raised by the study are discussed below.

Equity

TB often affects the most vulnerable in society such as those living in deprivation or experiencing the effects of substance misuse or time in prison.[16] It is important that we include these voices in our research. TB teams have been sensitised to identification of participants based on the eligibility criteria alone. If there are concerns about violence towards staff or difficulty engaging, participants will be offered remote interviews only.

TB affects migrant populations. Our approach to recruitment means that English speaking migrants from a range of backgrounds can be included. However, we have restricted the number of languages included and this means that some voices will not be heard.

For peer review only

We have made the decision to transcribe all interviews in the language of the participants to allow reporting in the source language and to ensure that all participants' original words are available for secondary analysis.

Recruitment

The duration of TB treatment means that clinical teams and patients may develop strong relationships. To reduce the risk of participants feeling obliged to participate, they will be made aware that non-participation will not negatively affect their care and that they are free to withdraw at any time. Although the clinical teams will identify potential participants, consent will be obtained by the research team.

Harms to participants

Some participants may find it distressing to talk about their experiences. Should this occur, the interview will be paused or terminated. FK is an experienced clinician and has training and experience in de-escalating situations. The nature of narrative interviews means that participants are in control of the level of detail they reveal. Participants will be allowed time to debrief and will be signposted to local support services if required.

Interviews will take place remotely or in clinical spaces and during normal working hours to limit potential environmental harms to both participants and researchers, and to allow prompt responses to expressed clinical needs. Input from our PPI partner suggested that evenings and weekends were valuable free time for those in employment and so should not be used for interviews. This approach may result in recruitment bias against those who are in full time employment. We will report the numbers of those who were unable to take part in the study due to this decision.

Confidentiality

Data will be stored in anonymised, encrypted, electronic formats. IT Security Teams have been consulted to ensure that data storage and transfer procedures are appropriate. Aspects of the narrative which might lead to identification of participants will not be reported.

The patient information leaflet clarifies that there may be situations in which disclosures are needed. In line with General Medical Council, UK, guidelines,[59] participants will first be asked for permission to disclose information. If permission is not forthcoming, then identifiable information will only be shared if this is in the interests of the patient or the public. All disclosures will be noted as part of the participants research record.

Stigma

Erving Goffman described three domains types of stigma, that associated with physical or mental illness (for example TB), that associated with identifying as part of a marginalised group (for example ethnic minorities or being an older adult), or that associated with moral character (for example substance misuse) (quoted in [60]). Our study participants are vulnerable to stigmatisation due to a confluence of one or more of these domains. Following guidelines suggested by Gabbidon and Chenneville, 2020, [60] we are reducing inadvertent stigmatisation by engaging in reflexivity around positionality and consulting with patient partners. Narrative methods give control of the content shared to study participants before allowing probing by the interviewer. We have clarified pathways for additional support should the interviews cause distress. We intend to describe the context and will take care to avoid identification through quotes. Participants will be anonymously acknowledged.

Infection control concerns

For peer review only - http://bmjopen.bmj.com/site/about/guidelines.xhtml

Face to face interviews will only take place after the TB team confirm that this is safe. The use of personal protective equipment and distancing measures will be determined by national guidelines, University and NHS Trust policies.

Case note reviews

Accessing case notes may make participants feel exposed or vulnerable. To this end, participants will be able to consent to the interview study alone.

Data extraction will be kept proportionate by using a pre-identified list of symptoms and signs or the participant's own words to identify appropriate material for the study.

Case note review may also make participants' GPs feel exposed. A graded approach will be taken to handling potential risks to participant safety. Where there is an ongoing or active risk, for example monitoring blood tests not being carried out, this will be flagged up using an auditable electronic messaging service.

Where there is the suggestion of historic risk to patient safety, for example avoidable treatment delay, the surgery will be made aware of this as a potential significant event. Preferred routes and recipients of each type of communication will be determined on a surgery-by-surgery basis.

Dissemination

This study forms part of a larger body work which will be submitted and published as a PhD thesis. Following analysis, a plain language summary will be shared with participants. Findings will be shared in peer reviewed journals and through public facing materials developed in conjunction with TB services, primary care colleagues, patient partners and TB charities.

FUNDING STATEMENT

FK is funded by the NIHR Doctoral Research Fellowship, grant number 300688. The views expressed are those of the author(s) and not necessarily those of the NIHR or the Department of Health and Social Care.

The project is being delivered with support from the Clinical Research Networks for Primary and Secondary Care in the West Midlands.

COMPETING INTERESTS STATEMENT

There are no competing interests to declare.

AUTHOR CONTRIBUTIONS

FK conceived the study with support from KS and NMc

FK wrote the first draft of the paper and all authors commented on revisions.

DATA STATEMENT SECTION

Interview transcripts will be stored for ten years after the end of the study and will made available for secondary analysis on a case-by-case basis.

ETHICS APPROVAL

For peer review only

The study was approved by the London - Harrow Research Ethics Committee. REC reference - 22//PR/0357. Protocol number SOC.06/20-21. IRAS ID 287971

REFERENCES

1. UK Health Security Agency, *Tuberculosis in England 2022 report (data up to the end of 2021).* 2022 June 2023 [cited 2023; Available from: https://www.gov.uk/government/publications/tuberculosis-in-england-2022-report-data-up-to-end-of-2021/tb-incidence-and-epidemiology-in-england-2021.
2. UK Health Security Agency, *TB Action Plan for England, 2021 to 2026*. 2021: London.
3. Greenaway, C., et al., *Delay in Diagnosis among Hospitalized Patients with Active Tuberculosis—Predictors and Outcomes.* American Journal of Respiratory and Critical Care Medicine, 2002. **165**(7): p. 927-933.
4. Virenfeldt, J., et al., *Treatment delay affects clinical severity of tuberculosis: a longitudinal cohort study.* BMJ Open, 2014. **4**.
5. Cheng, S., et al., *Effect of Diagnostic and Treatment Delay on the Risk of Tuberculosis Transmission in Shenzhen, China: An Observational Cohort Study, 1993–2010.* PLOS ONE, 2013. **8**(6): p. e67516.
6. Golub, J.E., et al., *Delayed tuberculosis diagnosis and tuberculosis transmission.* The International Journal of Tuberculosis and Lung Disease, 2006. **10**(1): p. 24-30.
7. Melsew, Y.A., et al., *Risk factors for infectiousness of patients with tuberculosis: a systematic review and meta-analysis.* Epidemiology and Infection, 2018. **146**(3): p. 345-353.
8. NICE. *NICE Guideline 33 - Tuberculosis*. 2016 27.08.2018]; Available from: https://www.nice.org.uk/guidance/ng33.
9. Nnoaham, K.E., et al., *Perceptions and experiences of tuberculosis among African patients attending a tuberculosis clinic in London.* The International Journal of Tuberculosis and Lung Disease, 2006. **10**(9): p. 1013-1017.
10. Public Health England, *Tuberculosis in England 2015 Report: presenting data to the end of 2014* 2015.
11. Public Health England, *Tuberculosis in England 2016 report: presenting data to end of 2015.* 2016, PHE.
12. Public Health England, *Tuberculosis in England 2017 report (presenting data to end of 2016).* 2017.
13. Public Health England, *Tuberculosis in England 2018 report. Presenting data to end of 2017.* 2018: PHE publications.
14. Public health England, *Tuberculosis in England 2019 report (presenting data to end of 2018).* 2019: PHE, London.
15. Public Health England, *Tuberculosis in England 2020 report presenting data to end of 2019.* 2020, PHE.
16. UK Health Security Agency, *Tuberculosis in England. 2021 report (Presenting data to end 2020)*. 2021, UKHSA: UK.
17. Broderick, C., et al., *Delays in the diagnosis and treatment of bone and joint tuberculosis in the United Kingdom.* The Bone & Joint Journal, 2018. **100-B**(1): p. 119-124.
18. Abbara, A., et al., *Time to diagnosis of tuberculosis is greater in older patients: a retrospective cohort review.* ERJ open research, 2019. **5**(4): p. 00228-2018.
19. Walter, F., et al., *The Andersen Model of Total Patient Delay: a systematic review of its application in cancer diagnosis.* Journal of health services research & policy, 2012. **17**(2): p. 110-118.
20. Wieland, M.L., et al., *Perceptions of tuberculosis among immigrants and refugees at an adult education center: a community-based participatory research approach.* Journal of immigrant and minority health, 2012. **14**(1): p. 14-22.

For peer review only

21. Gerrish, K., A. Naisby, and M. Ismail, *The meaning and consequences of tuberculosis among Somali people in the United Kingdom.* Journal of Advanced Nursing, 2012. **68**(12): p. 2654-2663.
22. Craig, G.M., A. Joly Lm Fau - Zumla, and A. Zumla, *'Complex' but coping: experience of symptoms of tuberculosis and health care seeking behaviours--a qualitative interview study of urban risk groups, London, UK.* BMC Public Health, (1471-2458 (Electronic)).
23. Quattrocchi, A., et al., *Determinants of patient and health system delay among Italian and foreign-born patients with pulmonary tuberculosis: a multicentre cross-sectional study.* BMJ Open, 2018. **8**.
24. Asch, S., et al., *Why Do Symptomatic Patients Delay Obtaining Care for Tuberculosis?* American Journal of Respiratory and Critical Care Medicine, 1998. **157**(4): p. 1244-1248.
25. Calder, L., W. Gao, and G. Simmons, *Tuberculosis: reasons for diagnostic delay in Auckland.* N Z Med J, 2000. **113**: p. 483.
26. Peri, A.M., et al., *Determinants of patient and health care services delays for tuberculosis diagnosis in Italy: a cross-sectional observational study.* BMC Infectious Diseases, 2018. **18**(1): p. 690.
27. Abarca Tomás, B., et al., *Tuberculosis in Migrant Populations. A Systematic Review of the Qualitative Literature.* PLOS ONE, 2013. **8**(12): p. e82440.
28. Roberts, D.J., et al., *Factors associated with delay in treatment initiation for pulmonary tuberculosis.* ERJ Open Research, 2020. **6**.
29. Wyman, M.F., S. Shiovitz-Ezra, and J. Bengel, *Ageism in the Health Care System: Providers, Patients, and Systems*, in *Contemporary Perspectives on Ageism*, L. Ayalon and C. Tesch-Römer, Editors. 2018, Springer International Publishing: Cham. p. 193-212.
30. Jones, D., et al., *Factors influencing symptom appraisal and help-seeking of older adults with possible cancer: a mixed-methods systematic review.* British Journal of General Practice, 2022. **72**(723): p. e702.
31. Teo, K., et al., *Help-Seeking Behaviors Among Older Adults: A Scoping Review.* Journal of Applied Gerontology, 2022. **41**(5): p. 1500-1510.
32. Paynter, S., et al., *Patient and health service delays in initiating treatment for patients with pulmonary tuberculosis: retrospective cohort study.* The International Journal of Tuberculosis and Lung Disease, 2004. **8**(2): p. 180-185.
33. Loutet, M.G., et al., *Delay from symptom onset to treatment start among tuberculosis patients in England, 2012–2015.* Epidemiology and Infection, 2018. **146**(12): p. 1511-1518.
34. Tattevin, P., et al., *Factors associated with patient and health care system delay in the diagnosis of tuberculosis in France.* The International Journal of Tuberculosis and Lung Disease, 2012. **16**(4): p. 510-515.
35. Saldana, L., et al., *Factors affecting delay in initiation of treatment of tuberculosis in the Thames Valley, UK.* (1476-5616 (Electronic)).
36. Farah, M.G., et al., *Patient and health care system delays in the start of tuberculosis treatment in Norway.* BMC infectious diseases, 2006. **6**: p. 33-33.
37. Sultan, H., S. Haroon, and N. Syed, *Delay and completion of tuberculosis treatment: a cross-sectional study in the West Midlands, UK.* Journal of Public Health, 2013. **35**(1): p. 12-20.
38. Alderson, P., *Critical realism for health and illness research: A practical introduction*. 2021: Policy Press.
39. Koopmans, E. and D.C. Schiller, *Understanding Causation in Healthcare: An Introduction to Critical Realism.* Qualitative Health Research, 2022. **32**(8-9): p. 1207-1214.
40. Greenhalgh, T. and B. Hurwitz, *Why study narrative?* BMJ, 1999. **318**: p. 48-50.
41. Smith B, *Narrative Analysis*, in *Analysing Qualitative Data in Psychology*, Lyons E and C. A, Editors. 2016, Sage: London.
42. Connelly, F.M. and D.J. Clandinin, *Stories of Experience and Narrative Inquiry.* Educational Researcher, 1990. **19**(5): p. 2-14.

For peer review only

43. Burck, C., *Comparing qualitative research methodologies for systemic research: the use of grounded theory, discourse analysis and narrative analysis.* Journal of Family Therapy, 2005. **27**(3): p. 237-262.
44. Hsu, M.Y. and B. McCormack, *Using narrative inquiry with older people to inform practice and service developments.* Journal of Clinical Nursing, 2012. **21**(5-6): p. 841-849.
45. Schwind, J.K., et al., *What can be learned from patient stories about living with the chronicity of heart illness? A narrative inquiry.* Contemporary Nurse, 2016. **52**(2-3): p. 216-229.
46. Sim, J., et al., *Can sample size in qualitative research be determined a priori?* International Journal of Social Research Methodology, 2018. **21**(5): p. 619-634.
47. Vasileiou, K., et al., *Characterising and justifying sample size sufficiency in interview-based studies: systematic analysis of qualitative health research over a 15-year period.* BMC Medical Research Methodology, 2018. **18**(1): p. 148.
48. Guest, G., A. Bunce, and L. Johnson, *How Many Interviews Are Enough?:An Experiment with Data Saturation and Variability.* Field Methods, 2006. **18**(1): p. 59-82.
49. Hennink, M.M., B.N. Kaiser, and V.C. Marconi, *Code Saturation Versus Meaning Saturation:How Many Interviews Are Enough?* Qualitative Health Research, 2017. **27**(4): p. 591-608.
50. Public Health England. *Tuberculosis: notifying cases*. 2014 2015 Jan 2021]; Available from: https://www.gov.uk/government/publications/tuberculosis-notifying-cases.
51. Jovchelovitch, S. and M.W. Bauer. *Narrative interviewing {online}*. originally published in Bauer, Martin W.; Gaskell, G.; Eds., Qualitative researching with text, image and sound : a practical handbook. London, England: SAGE Publications, 2000. 2000 15.05.2020]; Available from: http://eprints.lse.ac.uk/2633
52. Schmier, J.K. and M.T. Halpern, *Patient recall and recall bias of health state and health status.* Expert Review of Pharmacoeconomics & Outcomes Research, 2004. **4**(2): p. 159-163.
53. Haydon, G. and P. van der Riet, *Narrative inquiry: A relational research methodology suitable to explore narratives of health and illness.* Nordic Journal of Nursing Research, 2016. **37**(2): p. 85-89.
54. Lopez, G.I., et al., *Translation Barriers in Conducting Qualitative Research With Spanish Speakers.* Qualitative Health Research, 2008. **18**(12): p. 1729-1737.
55. Wong, J.P.-H. and M.K.-L. Poon, *Bringing Translation Out of the Shadows: Translation as an Issue of Methodological Significance in Cross-Cultural Qualitative Research.* Journal of Transcultural Nursing, 2010. **21**(2): p. 151-158.
56. Emden, C., *Conducting a narrative analysis.* Collegian, 1998(1322-7696 (Print)).
57. Farmer, T., et al., *Developing and Implementing a Triangulation Protocol for Qualitative Health Research.* Qualitative Health Research, 2006. **16**(3): p. 377-394.
58. Greenhalgh, T., *Cultural contexts of health: the use of narrative research in the health sector*, in *Health Evidence Network (HEN) synthesis report*. 2016, WHO Regional Office for Europe: Copenhagen.
59. Council, G.M. *Ethical and legal duties of confidentiality*. 2020 30.07.2020]; Available from: https://www.gmc-uk.org/ethical-guidance/ethical-guidance-for-doctors/confidentiality/disclosures-for-the-protection-of-patients-and-others.
60. Gabbidon, K. and T. Chenneville, *Strategies to minimize further stigmatization of communities experiencing stigma: A guide for qualitative researchers.* Stigma and Health, 2021. **6**(1): p. 32-42.

For peer review only

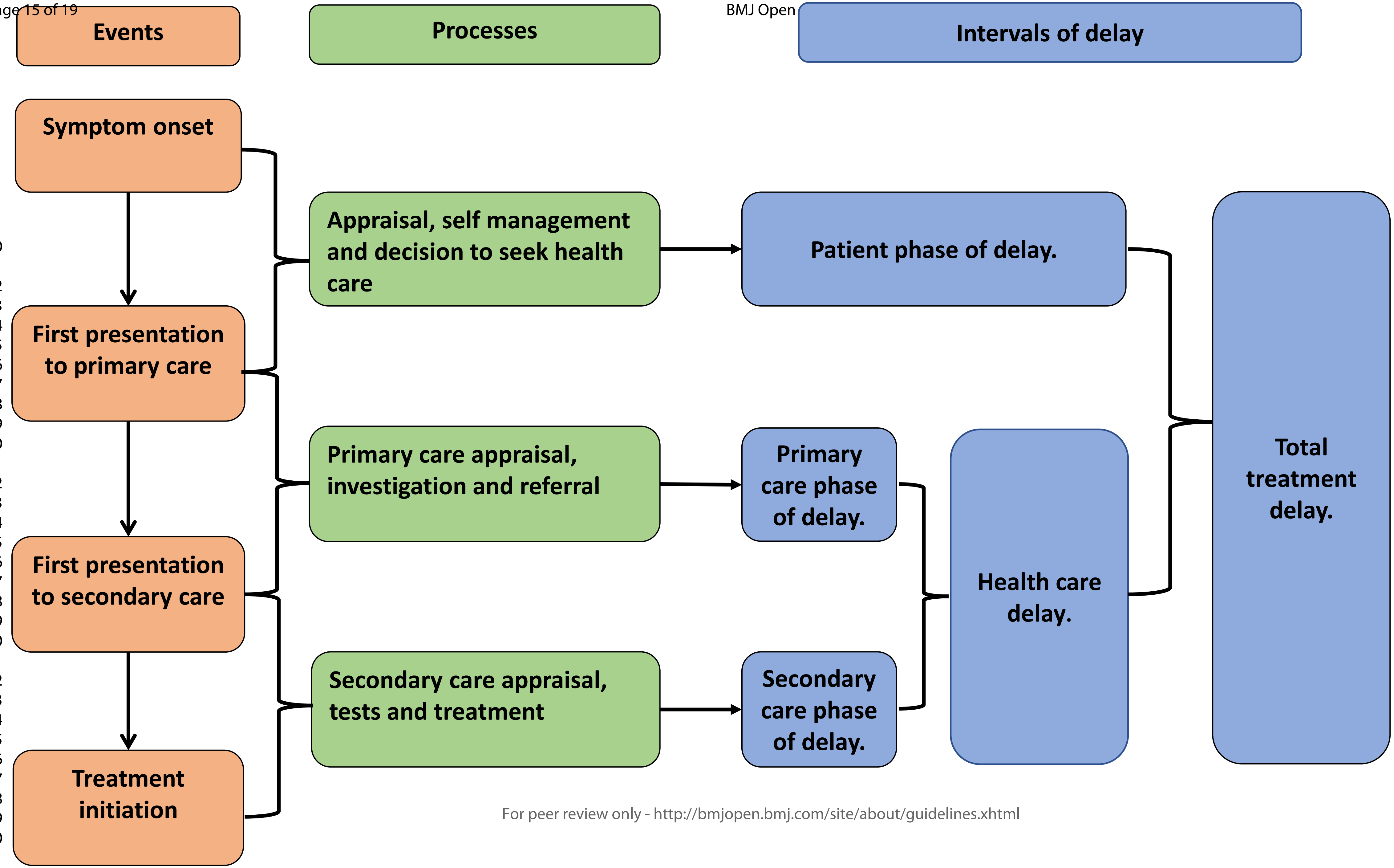
Events
Processes
Intervals of delay
Symptom onset
Appraisal, self management and decision to seek health care
Patient phase of delay.
First presentation to primary care
Primary care appraisal, investigation and referral
Primary care phase of delay.
Total treatment delay.
Health care delay.
First presentation to secondary care
Secondary care appraisal, tests and treatment
Secondary care phase of delay.
Treatment initiation

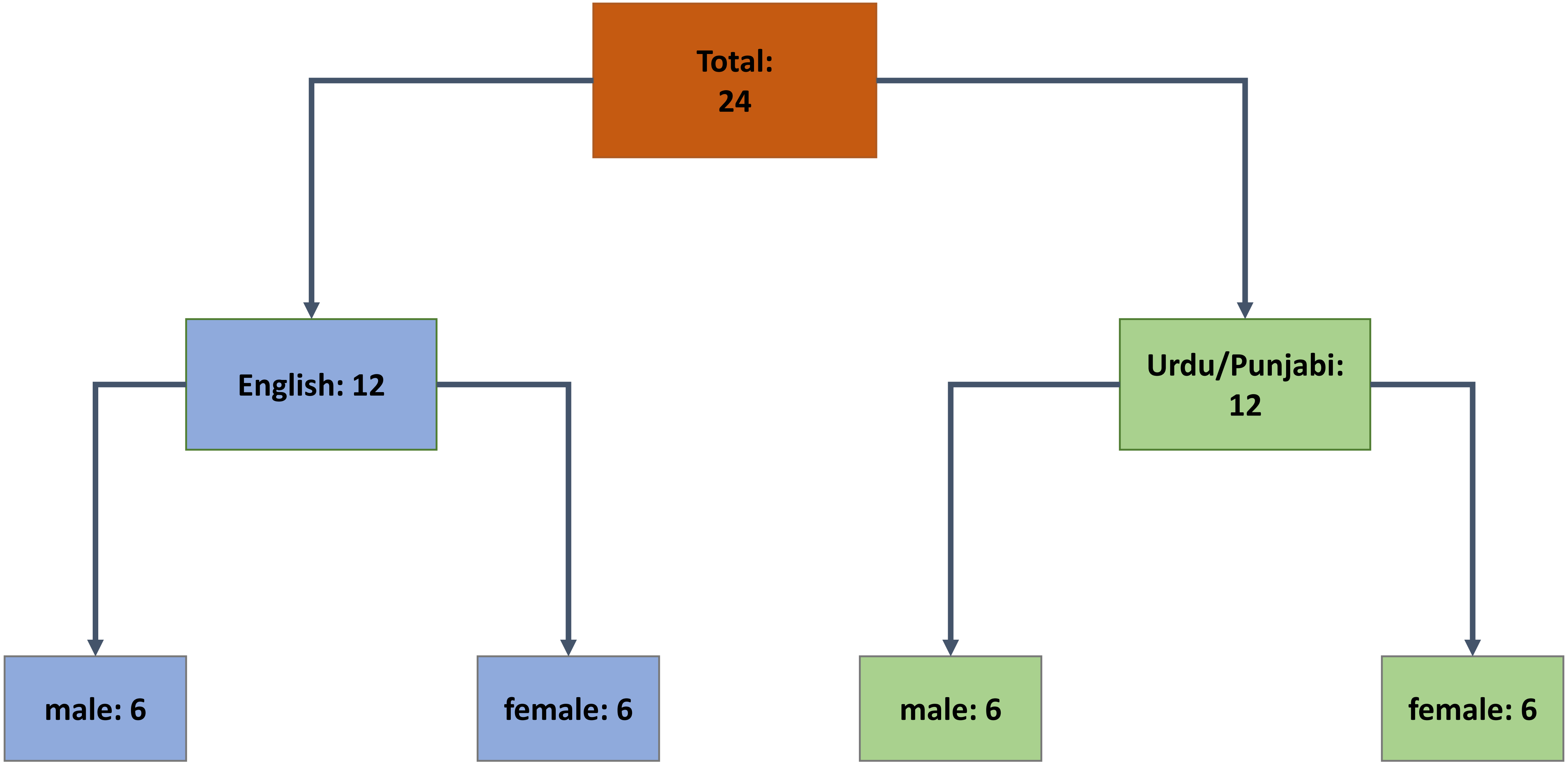
Total:
24
English: 12
Urdu/Punjabi:
12
male: 6
female: 6
male: 6
female: 6

Supplementary Material

From symptom onset to treatment initiation: A narrative study of Older Adults with Tuberculosis (OAT)

The OAT Study

Topic Guide

(Date 20/09/2020 Version 1.0)

For peer review only

The OAT Study will take a narrative approach to data gathering. The guide will the trialled on English and non-English speaking participants and adapted if needed.

Step One: Introductions, consent and identity confirmation

- Introduce self and confirm that the call is to take consent and carry out the interview.
- Ask participant to confirm their name and date of birth.
- Confirm that this is a suitable time.
- Ask participant if they have any questions about the information sheets, consent form or study in general.
- Thanks for agreeing to participate.
- Advise re likely duration: about one hour
- Check if there is anyone else in the room. Confirm that the participant is happy for that person to be in room. Ask that others in the room do not interject during the interview and advise them that it may not be possible to remove their comments from the recordings, but that these comments will not be used for the research. Be prepared to comment during the interview if there are too many interruptions.
- Explain the format of the interview – ie
    - participant invited to tell their story.
    - Define the scope – from time of onset of illness to treatment initiation.
    - "Once you have finished telling your story, I might ask you some questions."
- Advise the participant that recording will begin and turn recording devices on.
- State
    - "This is the audio recorded consent for the OAT Study".
    - For "PARTICPANT NAME" and "DATE OF BIRTH".
    - Recorded on "DATE" by "INVESTIGATOR'S NAME".
    - "I am going to read each statement from Consent Form Two. If you agree with the statement, please say "I agree". You must agree with all the statements to be eligible to take part in the study. Your consent will be audio recorded and stored securely."
    - Read each statement from Consent Form Two. Ensure that participant's agreement to each statement is clearly heard.

For peer review only - http://bmjopen.bmj.com/site/about/guidelines.xhtml

Step Two: Initiation of the interview and main narration

- Advise participant that interview will begin
- "I am interested in trying to understand your experience of being diagnosed with TB. Can you tell me what happened and how you felt from the time you first became unwell until you started treatment for TB?"
- Take notes and use verbal and non-verbal prompts.
- When the narrative reaches a natural end, ask, "Is there something else you want to tell me?"

Step Three: Questioning Phase.

Questions should be formulated using the participant's own words. The questions below are a guide only.

Questions should not start with "Why" and should not be used to point out contradictions in the narrative.

Points which should be probed if they have not been covered in the narrative. Every point will not be relevant to all participants:

- Generally applicable question structure, "You mentioned XXX, can you say a bit more about that?"
- Is symptom onset clearly identified. For example, "How were you feeling before "FIRST SYMPTOMS" started?"
- Appraisal process. For example, "What made you realise that you were not well?"
- Symptoms before presenting to health care. For example, "How were you feeling before you went to see the doctor or nurse?"
- Trigger to seek health care. For example, "What made you decide to go and see the doctor or nurse?"
- Influence of others on the decision to seek health care. For example, "Did friends or family encourage or discourage you from seeing a doctor or nurse?"
- Ease of access to health care. For example, "Was it easy to get an appointment to see a doctor or nurse?"
- Experience of first and subsequent contacts with health care. For example, "What happened when you first saw a doctor or nurse?" and/ or, "How did you feel after you went to see the doctor or nurse?"
- Experience of waiting for a diagnosis. For example, "How did you feel while you were waiting for a diagnosis?"
- Experience of getting a diagnosis. For example, "What did it feel like when you found out you have TB?"
- Experience of receiving treatment. "Can you tell me more about the experience of receiving treatment?"
- Impact of COVID. "How did COVID affect you and your care?"

Personal details – if not captured in the interview

- Employment status
- Housing situation
- Country of birth

For peer review only

Step Four: Concluding talk

- Ask participant if they are happy with the information shared
- Advise the participant that the recording is ending.
- Note additional information received after the recording is completed as contextual information in field notes. This will not be included in the analysis.

For peer review only
